# Ruxolitinib mediated paradoxical JAK2 hyperphosphorylation is due to the protection of activation loop tyrosines from phosphatases

**DOI:** 10.1038/s41375-025-02594-7

**Published:** 2025-04-23

**Authors:** Sivahari P. Gorantla, Lorenz Oelschläger, Gerin Prince, Jasmin Osius, Suresh Babu Kolluri, Yamil Maluje, Anke Fähnrich, Nancy Ernst, Alanis Barbosa Gulde, Ralf Joachim Ludwig, Timo Gemoll, Stephanie Fliedner, Wencke Walter, Torsten Haferlach, Niklas Gebauer, Hauke Busch, Justus Duyster, Nikolas von Bubnoff

**Affiliations:** 1 Department of Hematology and Oncology, University Medical Center Schleswig-Holstein, and University Cancer Center Schleswig-Holstein, Lübeck, Germany; 2 Institute of Experimental Dermatology, University of Luebeck, Luebeck, Germany; 3 Section for Translational Surgical Oncology & Biobanking, Department of Surgery, University Medical Center Schleswig-Holstein, Campus Lübeck & University of Luebeck, Luebeck, Germany; 4 MLL Münchner Leukämielabor GmbH, Max-Lebsche-Platz 31, 81377 München, Germany; 5 Department of Internal Medicine I, University Medical Center Freiburg, Hugstetter Str.55, 79106 Freiburg, Germany

**Keywords:** Myeloproliferative disease, Myeloproliferative disease

## Abstract

Myelofibrosis (MF) in 50% of cases is driven by an activating JAK2 mutation, mostly V617F. Ruxolinitib is approved for the treatment of MF. Responses to ruxolitinib in MF are of limited duration. Unexpectedly, treatment of JAK2-V617F expressing cells with ruxolitinib causes paradoxical hyperphosphorylation of JAK2 at activation loop Tyr1007/Tyr1008. The significance of ruxolitinib-induced JAK2 hyperphosphorylation is not well understood. We found that a ruxolitinib-resistant JAK2 variant (V617F + L983F) and a kinase dead mutant (JAK2-V617F + K882R) did not show paradoxical hyperphosphorylation after ruxolitinib treatment indicating that it is an intrinsic mechanism. Antibodies against pTyr1007/1008 failed to immunoprecipitate native JAK2-V617F in the presence of ruxolitinib, although JAK2-V617F was hyperphosphorylated at these sites, suggesting that in the presence of ruxolitinib the JAK2 activation loop is buried within the kinase domain. This stabilization of the activation loop conformation resulted in the protection of pTyr1007/1008 sites from phosphatases. Mutation of Arg975 and Lys999 to Ala reduced the phosphorylation at both Tyr1007/Tyr1008 residues, and notably, ruxolitinib treatment did not lead to JAK2 hyperphosphorylation. Importantly, hyperphosphorylated JAK2 after ruxolitinib dissociation displayed excess rebound activation of STAT5 target gene PIM kinase. Our results suggest a novel mode of kinase regulation by modulating kinase activity through conformational changes induced by ruxolitinib.

Subject categories: JAK2-V617F, Ruxolitinib, JAK2 hyperphosphorylation, Phosphatases action, PIM kinases

## Introduction

Myeloproliferative neoplasms (MPNs) such as polycythemia vera (PV), essential thrombocythemia (ET) and primary myelofibrosis (PMF), are frequently associated with the somatic V617F mutation in the pseudo kinase domain of JAK2 [1–4]. Several JAK2 inhibitors, including ruxolitinib, fedratinib, and momelotinib showed remarkable clinical activity in primary and secondary myelofibrosis (MF) patients [5–9]. Among these, ruxolitinib is the first JAK inhibitor approved by the FDA for the treatment of MF and PV. Phase III studies demonstrated the superiority of ruxolitinib over placebo for reduction of spleen size and hematological response [10, 11]. In the COMFORT-I trial, MF patients treated with ruxolitinib displayed spleen response duration of 168.3 weeks and prolonged median overall survival versus placebo. In a phase 1/2 trial, the median survival after ruxolitinib discontinuation was 14 months, underscoring the need for improved therapies [12]. Despite very good clinical response, the impact of ruxolitinib on the abnormal hematopoietic clone is limited (“persistence”) [13]. The mechanism of ruxolitinib persistence is unclear. Notably, it has been demonstrated that treatment of ruxolitinib did not decrease phosphorylation of activation loop Tyr1007/Tyr1008 as one would presume, but rather a paradoxical increase [14].

Type I JAK2 inhibitors such as ruxolitinib, fedratinib and lestaurtinib bind the JAK2 in active conformation when activation loop is fully phosphorylated. In contrast to type I inhibitors, type II mode of inhibition of JAK2 by CHZ868 does not lead to paradoxical increase in phosphorylation of activation loop Tyr1007/Tyr 1008 [15, 16]. In this study, we aimed to identify the mechanism of paradoxical hyperphosphorylation of JAK2 activation loop tyrosines in the presence of ruxolitinib. Our data suggest that ruxolitinib binding stabilizes the conformation of the activation loop within the kinase domain with the help of Arg975 and Lys999, leading to the protection of the activation loop tyrosines from the action of phosphatases. In addition, we found that hyperphosphorylated JAK2, once released from bound ruxolitinib can hyperactivate STAT5 and PIM kinases.

## Materials and methods

### Inhibitors and cytokines

Ruxolitinib was provided by Novartis Pharma AG, Basel, Switzerland. Fedratinib was purchased from Selleckchem, Houston, USA. Lestaurtinib was purchased from Calbiochem. All the inhibitors were dissolved in dimethyl sulfoxide (DMSO) to make stock solutions of 10 mM and stored at –20 °C. Mouse IL-3 was purchased from R&D systems. Cytokine stock solutions were prepared and used 20 ng/ml for stimulation experiments.

### Antibodies

The anti-phosphotyrosine antibody was purchased from Upstate Biotechnology (4G10) (Biozol, Eching, Germany) and Transduction (PY20) (BD Biosciences). STAT5 (G-2), ID1, PIM1, PIM2 pJAK2 (21870-R) antibodies were obtained from Santacruz Biotechnology (Heidelberg, Germany). JAK2 c-terminal antibody (D2E12 XP^R^), pSTAT5, pTYK2, TYK2, pJAK3, JAK3, pAkt, pERK, ERK, c-Myc, BCL-2, Akt were purchased from cell signaling.

### Proliferation assay

Proliferation was measured using an MTS (3-(4,5 dimethylthiazol-2-yl)-5-(3-carboxymethoxyphenyl-2-(4-sulfophenyl)-2H-tetrazolium))-based method by absorption of formazan at 490 nm (CellTiter 96; Promega, Madison, WI). Measurements were taken in triplicate after 72 and 96 hours of culture without cytokines, as described previously [17].

### Statistical analysis

Values are represented as mean $\pm \,$SEM. The comparison of multiple groups was analyzed by a one-way ANOVA test, and the comparison between two groups was analyzed by unpaired *t* test. **p < 0.01, *p < 0.05 and n.s., not significant, *p* > 0.05 by Student’s *t* test. **p < 0.005, ****p < 0.0001 were considered for one-way ANOVA test.

The remaining materials and methods are in the supplementary information file.

## Results

### Type I JAK2 inhibitors paradoxically promote tyrosine phosphorylation of JAK2 activation loop tyrosines

**Fig. 1:**
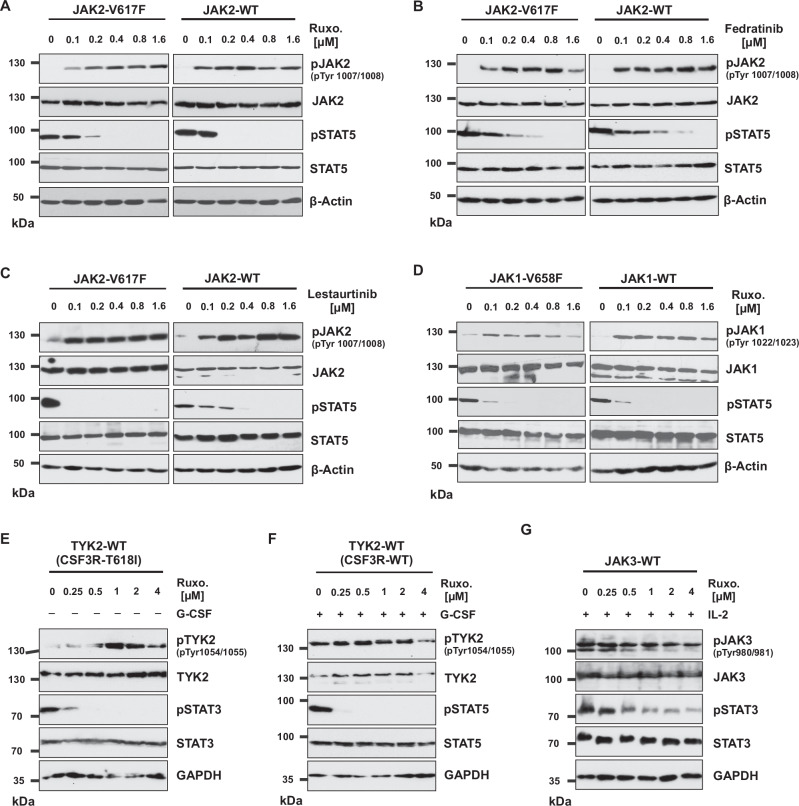
JAK2 ATP-competitive inhibitors treatment leads to paradox JAK2 hyperphosphorylation. Ba/F3 cells expressing the JAK2-V617F and JAK2-WT were treated with the indicated concentration of the ruxolitinib (**A**) fedratinib (**B**) and lestaurtinib (**C**) for 3 h, and lysates were prepared and subjected to western blotting for the pJAK2, JAK2, pSTAT5 and STAT5. Ba/F3 cells expressing the JAK1-V658F and JAK1-WT were treated for 3.5 h with ruxolitinib, and lysates were analyzed for activation of JAK1 and STAT3 (**D**). Ba/F3 cells stably expressing the CSF3R-T618I and CSF3R-WT treated with indicated concentrations of ruxolitinib (**E**) and G-CSF (**F**) for 3 h, and lysates were subjected to western blotting for the pTYK2, TYK2, pSTAT3, STAT3, pSTAT5 and STAT5. Ba/F3 cells expressing the JAK3-WT were treated with indicated concentrations of ruxolitinib in the presence of interleukin-2 (IL-2), and lysates were subjected to western blotting for the pJAK3, JAK3, pSTAT3 and STAT3 (**G**).

Ruxolitinib is a potent JAK1/JAK2 specific inhibitor exhibiting remarkable clinical activity in JAK2-V617F mediated MPNs [18]. However, JAK inhibitors do not have a major impact on the abnormal hematopoietic clone, and responses, especially in MF, are not durable. Responses have been linked to suppression of inflammatory cytokines [5]. However, the mechanism leading to disease persistence in MF patients receiving JAK1/2 inhibitors is not fully understood. To explore this, we performed a western blot analysis of JAK2-V617F expressing Ba/F3 cells pre-treated with ruxolitinib. Ruxolitinib treatment inhibited phosphorylation of STAT5 but paradoxically increased Tyr1007/Tyr1008 phosphorylation of the JAK2 activation loop (Fig. 1A) as shown before [19]. To investigate whether ruxolitinib specifically induces JAK2 hyperphosphorylation in the presence of the JAK2-V617F mutation, we treated Ba/F3 cells expressing WT-JAK2 with ruxolitinib. Like JAK2-V617F, JAK2-WT also showed an increase in Tyr1007/Tyr1008 phosphorylation of the JAK2 activation loop and an inhibition of STAT5 phosphorylation (Fig. 1A). These results suggest that ruxolitinib treatment leads to hyperphosphorylation of JAK2 irrespective of mutational status. Subsequently, we tested whether paradoxical JAK2 hyperphosphorylation is exclusively mediated by ruxolitinib. We subjected Ba/F3 cells expressing either JAK2-V617F or JAK2-WT to the alternate type I ATP-competitive inhibitors (fedratinib and lestaurtinib), resulting in the same observation (Fig. 1B, C). This suggests that this pattern is a general effect of JAK2 ATP-competitive inhibitors. Next, we treated with next-generation type I ATP-competitive inhibitors of JAK2, such as BMS-911543 and momelotinib, and found paradoxical activation of the JAK2 activation loop Tyr1007/Tyr1008 similar to ruxolitinib (Supplementary Fig. S1A, B). Finally, similar results were obtained in HEL (JAK2-V617F positive) cells upon treatment with ruxolitinib, suggesting that our observations are not cell-type specific (Supplementary Fig. S1C). Since ruxolitinib is an inhibitor for both JAK1 and JAK2, we evaluated the JAK1 phosphorylation status in the presence of ruxolitinib. We treated Ba/F3 cells expressing constitutive active JAK1-V658F and JAK1-WT with ruxolitinib. JAK1 was also hyperphosphorylated in the residues Tyr1022/Tyr1023 of the activation loop, corresponding to Tyr1007/Tyr1008 in JAK2 (Fig. 1D).

Furthermore, we analyzed the TYK2 activation loop Tyr 1054/Tyr1055 phosphorylation after ruxolitinib treatment from constitutive active CSF3R-T618I and CSF3R-WT expressing Ba/F3 cells. Ruxolitinib paradoxically increased Tyr1054/Tyr1055 phosphorylation of the TYK2 activation loop (Fig. 1E, F). However, ruxolitinib treatment of JAK3 expressing Ba/F3 cells led to decrease in phosphorylation of JAK3 activation loop Tyr980/981 (Fig. 1G). Our findings suggest that JAK1 and TYK2 behave similarly to JAK2 but differ from JAK3, as previously observed [20] (Fig. 1G and Supplementary Fig. S1D). Structural analysis of activation loop tyrosines of JAK2 and JAK3 bound to CMP-6 (pan JAK-kinase inhibitor) revealed different folding of the activation loop between JAK2 and JAK3 (Supplementary Fig. S2). Taken together, our data suggest that type I JAK2 inhibitors promote paradoxical hyperphosphorylation of JAK1, JAK2 and TYK2 but not JAK3.

#### Ruxolitinib induces JAK2 hyperphosphorylation by an intrinsic mechanism

In order to rule out the possibility of catalytic activity of the JH2 domain in ruxolitinib-induced JAK2 hyperphosphorylation, a phospho-deficient mutant (S523A) and a loss of ATP binding mutant (K581R) were introduced to prevent the kinase activity of the JH2 domain. These variants, similarly to JAK2-V617F, showed paradoxical hyperphosphorylation of the JAK2 activation loop and inhibition of STAT5. (Supplementary Fig. S3 and Supplementary Information).

**Fig. 2:**
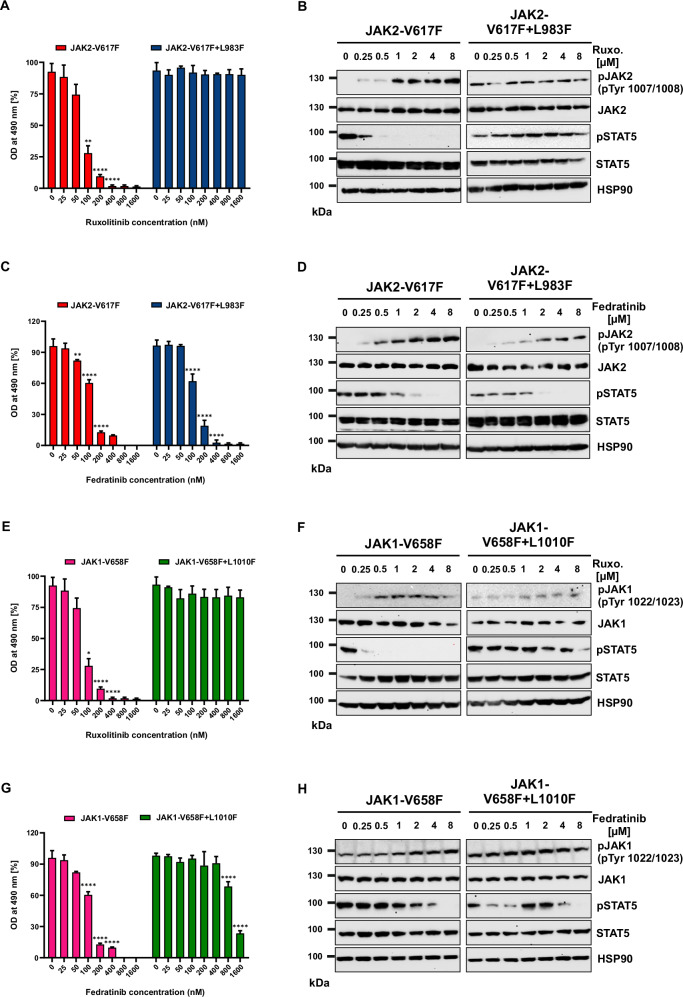
Ruxolitinib induced paradox JAK2 hyperphosphorylation is an intrinsic mechanism. JAK2-V617F and JAK2-V617F + L983F were stably expressed in Ba/F3 cells and analyzed for ruxolitinib sensitivity with the indicated concentrations. Proliferation was measured using 3-(4,5-dimethylthiazol-2-yl)-5-(3-carboxymethoxyphenyl)-2-(4-sulfophenyl)-2h-tetrazolium (MTS)- based method after incubation for 48 h in the presence of increasing concentrations of the inhibitor ruxolitinib (**A**). Data is shown as mean ± standard deviation (SD) (*n* = 3). OD—optical density. Immunoblot analysis of Ba/F3 cells expressing JAK2 mutants cultured with indicated concentrations of ruxolitinib for 4 h and lysates were subjected to indicated antibodies (**B**). A representative image of *n* = 2 two independent experiments is shown. JAK2-V617F and JAK2-V617F + L983F were stably expressed in Ba/F3 cells and analyzed for fedratinib sensitivity with the indicated concentrations. Proliferation was measured using 3-(4,5-dimethylthiazol-2-yl)-5-(3-carboxymethoxyphenyl)-2-(4-sulfophenyl)-2h-tetrazolium (MTS)- based method after incubation for 48 h in the presence of increasing concentration of the inhibitor fedratinib (**C**). Data is shown as mean ± standard deviation (SD) (*n* = 3). OD—optical density. Immunoblot analysis of Ba/F3 cells expressing JAK2 mutants cultured with indicated concentrations of fedratinib for 3 h and lysates were subjected to indicated antibodies (**D**). A representative image of *n* = 2 two independent experiments is shown. Ba/F3 cells expressing JAK1-V658F and JAK1-V658F + L1010F with ruxolitinib and measured the ruxolitinib sensitivity with the indicated concentrations. Proliferation was measured using 3-(4,5-dimethylthiazol-2-yl)-5-(3-carboxymethoxyphenyl)-2-(4-sulfophenyl)-2h-tetrazolium (MTS)- based method after incubation for 48 h in the presence of increasing concentrations of the inhibitor ruxolitinib (**E**). Data is shown as mean ± standard deviation (SD) (*n* = 3). OD—optical density. Immunoblot analysis of Ba/F3 cells expressing JAK1 mutants cultured with indicated concentrations of ruxolitinib for 3 h and lysates were subjected to indicated antibodies (**F**). A representative image of *n* = 2 two independent experiments is shown. Similarly, Ba/F3 cells expressing the JAK1 variants with the indicated concentration of fedratinib and measured the fedratinib sensitivity with the indicated concentrations. Proliferation was measured using 3-(4,5-dimethylthiazol-2-yl)-5-(3-carboxymethoxyphenyl)-2-(4-sulfophenyl)-2h-tetrazolium (MTS)- based method after incubation for 48 h in the presence of increasing concentration of the inhibitor fedratinib (**G**). Data is shown as mean ± standard deviation (SD) (*n* = 3). OD—optical density. Immunoblot analysis of Ba/F3 cells expressing JAK1 mutants cultured with increasing concentrations of fedratinib (0, 250, 500, 1000, 2000, 4000 and 8000 nM) for 4 h and lysates were subjected to indicated antibodies (**H**). A representative image of *n* = 2 two independent experiments is shown. ****p < 0.0001; **p < 0.01, *p < 0.05 and n.s., not significant, *p* > 0.05 by one-way ANOVA test.

Next, we hypothesized that in the presence of ruxolitinib, protein tyrosine phosphatases might be dissociated from the active JAK2 complex, resulting in increased phosphorylation of JAK2 Tyr1007/Tyr1008. It has been reported that protein tyrosine phosphatase (SHP-2) is a candidate phosphatase to dephosphorylate JAK2 on both Tyr1007/Tyr1008 [21]. We immunoprecipitated JAK2 from JAK2-V617F expressing cells with and without ruxolitinib treatment to analyze this possibility. The presence of ruxolitinib did not alter SHP-2 binding to JAK2 (Supplementary Fig. S4), suggesting that ruxolitinib-bound conformation of JAK2 does not lead to the dissociation of SHP-2 from the JAK2 complex. To investigate whether ruxolitinib-induced JAK2 hyperphosphorylation is a JAK2-extrinsic mechanism, we introduced a JAK2 kinase-dead mutant, which can bind to ruxolitinib but does not enfold intrinsic kinase activity (V617F + K889R). Notably, the presence of JAK2 kinase dead mutant treatment with ruxolitinib did not lead to hyperphosphorylation of the activation loop Tyr1007/Tyr1008, indicating that no upstream kinase was involved in ruxolitinib-induced JAK2 hyperphosphorylation (Supplementary Fig. S5). To test whether hyperphosphorylation is a JAK2-intrinsic mechanism, we created a ruxolitinib-insensitive variant (JAK2-V617F + L983F), we previously identified in ruxolitinib-resistant cell lines [16]. Ruxolitinib treatment in Ba/F3 cells expressing this variant failed to induce hyperphosphorylation of JAK2 activation loop Tyr1007/Tyr1008 (Fig. 2A, B) and did not suppress STAT5 phosphorylation and cell growth. In contrast, cells expressing the JAK2-V617F + L983F variant were sensitive towards fedratinib and induced Tyr1007/Tyr1008 hyperphosphorylation (Fig. 2C, D). These results suggest that ruxolitinib binding to the active kinase is required to induce hyperphosphorylation of the activation loop Tyr1007/Tyr1008. To confirm this possibility in JAK1, we cloned ruxolitinib-resistant homologous mutation L1010F in JAK1. As shown with JAK2, the ruxolitinib-insensitive JAK1-V658F + L1010F variant failed to induce Tyr1022/Tyr1023 hyperphosphorylation of JAK1 (Fig. 2E, F). Fedratinib suppressed STAT5 phosphorylation and cell proliferation, and induced Tyr1022/Tyr1023 hyperphosphorylation of JAK1 (Fig. 2G, H). Collectively, these results suggest that ruxolitinib-induced JAK2-V617F hyperphosphorylation is mediated by an intrinsic mechanism where ruxolitinib binding to JAK2 in its active conformation is required.

#### Ruxolitinib-induced JAK2 hyperphosphorylation is due to protection of activation loop tyrosines from phosphatases

**Fig. 3:**
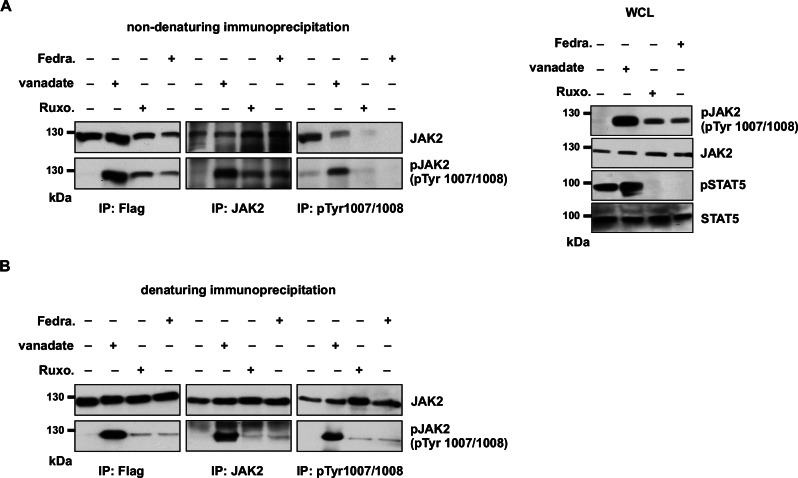
Phospho-specific antibody fail to immunoprecipitate JAK2 in the presence of ATP-competitive inhibitors. JAK2-V617F expressing Ba/F3 cells were treated without and with vanadate, ruxolitinib and fedratinib. Cells were lysed in a non-denaturing lysis buffer, and immunoprecipitation was carried out using the anti-flag antibody, anti-JAK2 antibody and phosphospecific Tyr1007/Tyr1008 JAK2 antibody. Immunocomplexes and whole-cell lysate (WCL) were analyzed by IB (**A**). Denaturing immunoprecipitation was carried out using the same antibodies mentioned in non-denaturing immunoprecipitation, and these results showed all the antibodies were able to immunoprecipitate JAK2 equally either in the presence or in the absence of ATP-competitive inhibitors (**B**). A representative image of *n* = 2 two independent experiments is shown.

We hypothesized that phosphorylation of JAK2 activation loop tyrosines accumulates in the presence of ruxolitinib due to reduced dephosphorylation of JAK2 by phosphatases. We therefore co-treated cells expressing JAK2-V617F with phosphatase inhibitor (vanadate) and ruxolitinib and measured the phosphorylation of activation loop Tyr1007/Tyr1008. When phosphatase activity was blocked, STAT5 phosphorylation, as expected, was not suppressed in the presence of ruxolitinib and JAK2 was strongly phosphorylated without ruxolitinib. Surprisingly, JAK2 Tyr1007/Tyr1008 phosphorylation was inhibited in the presence of ruxolitinib (Supplementary Fig. S6 left side panel). Removing the phosphatase inhibitor from the same cells led to a regain of the ruxolitinib-mediated JAK2 hyperphosphorylation of activation loop Tyr1007/Tyr1008 (Supplementary Fig. S6 right side panel). These results suggest involvement of phosphatases in this process. In the presence of ruxolitinib the activation loop might not be accessible to the phosphatases. Next, we hypothesized that in the presence of Type I inhibitors, the JAK2 activation loop is buried inside the kinase domain, which subsequently leads to the protection of the activation loop tyrosines 1007/1008 from phosphatases. To test this hypothesis, we performed native, non-denaturing immunoprecipitation in the presence and absence of ATP-competitive inhibitors. Equal amounts of JAK2 were immunoprecipitated with flag-tag (N-terminus) antibody in the presence of vanadate, ruxolitinib, fedratinib, and in untreated control cells (Fig. 3A left four lanes). Immunoprecipitation with JAK2 antibody recognizing the pseudokinase domain of JAK2 also immunoprecipitated equal amounts of JAK2 (Fig. 3A middle four lanes). In contrast, immunoprecipitation with a phospho-specific antibody (pY1007/1008) revealed that ruxolitinib and fedratinib treatment led to the failure of immunoprecipitation of JAK2 (Fig. 3A, left panel, right four lanes) even though JAK2 was hyperphosphorylated (Fig. 3A, right panel). However, untreated control and vanadate treatment still resulted in JAK2 immunoprecipitation. Importantly, denaturing immunoprecipitation rescued JAK2 immunoprecipitation with a phospho-specific antibody (pY1007/1008) in the presence of ruxolitinib and fedratinib (Fig. 3B). These results imply that the activation loop conformation is stabilized inside the kinase domain in the presence of ATP-competitive inhibitors, which likely protects the activation loop pTyr1007/Tyr1008 from phosphatases.

#### Destabilization of the activation loop conformation leads to a decrease in activation loop phosphorylation and prevents paradox JAK2 hyperphosphorylation

**Fig. 4:**
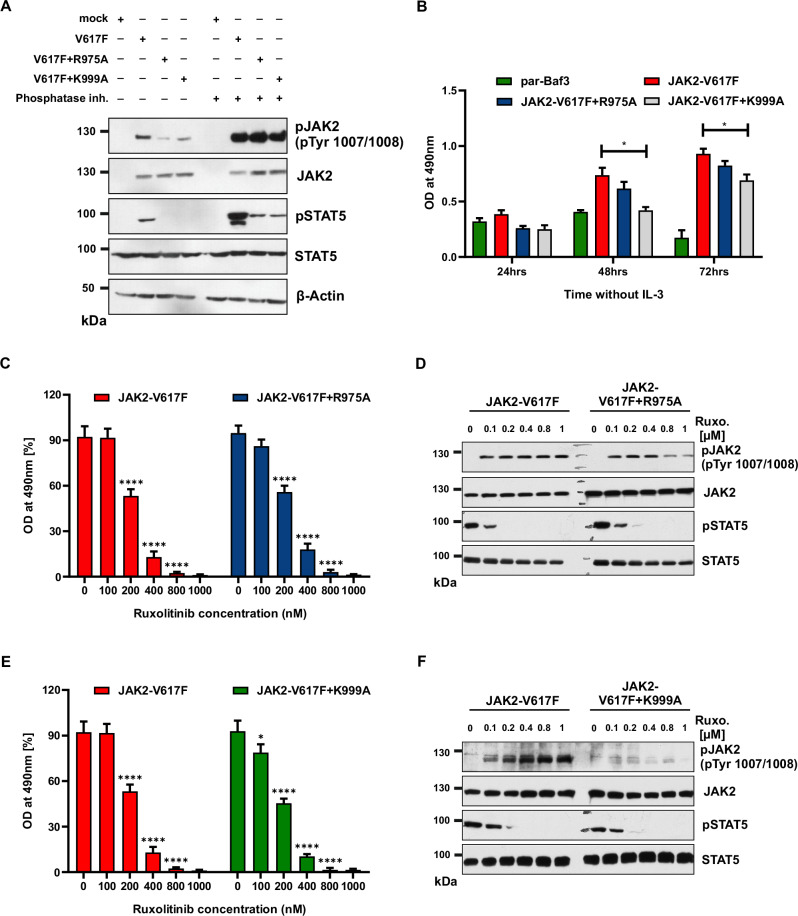
Destabilization of activation loop conformation leads to decrease in JAK2 activation and paradox JAK2 hyperphosphorylation. HEK293Tcells expressing the JAK2-V617F, JAK2-V617F + R975A and JAK2-V617F + K999A were serum starved and treated with 1 μM pervanadate and without and lysates were subjected with indicated antibodies (**A**). The proliferation of parental Ba/F3 cells and Ba/F3 cells expressing JAK2-V617FJAK2, JAK2-V617F + R975A and JAK2-V617F + K999A in the absence of interleukin-3 (IL-3) was quantified by the relative optical density (OD) after 96 h using MTS-based assay (**B**). Data is shown as mean ± standard deviation (SD) (*n* = 3). OD—optical density. JAK2-V617F and JAK2-V617F + R975A were stably expressed in Ba/F3 cells and analyzed for ruxolitinib sensitivity with the indicated concentrations. Proliferation was measured using 3-(4,5-dimethylthiazol-2-yl)-5-(3-carboxymethoxyphenyl)-2-(4-sulfophenyl)-2h-tetrazolium (MTS)- based method after incubation for 48 h in the presence of increasing concentrations of the inhibitor ruxolitinib (**C**). Data is shown as mean ± standard deviation (SD) (*n* = 3). OD—optical density. Immunoblot analysis of Ba/F3 cells expressing JAK2 mutants cultured with indicated concentrations of ruxolitinib for 4 h and lysates were subjected to indicated antibodies (**D**). A representative image of *n* = 2 two independent experiments is shown. JAK2-V617F and JAK2-V617F + K999A were stably expressed in Ba/F3 cells and analyzed for ruxolitinib sensitivity with the indicated concentrations. Proliferation was measured using 3-(4,5-dimethylthiazol-2-yl)-5-(3-carboxymethoxyphenyl)-2-(4-sulfophenyl)-2h-tetrazolium (MTS)- based method after incubation for 48 h in the presence of increasing concentration of the inhibitor ruxolitinib (**E**). Data is shown as mean ± standard deviation (SD) (*n* = 3). OD—optical density. Immunoblot analysis of Ba/F3 cells expressing JAK2 mutants cultured with increasing concentrations of ruxolitinib (0, 100, 200, 400, 800, and 1000 nM) for 4 h, and lysates were subjected to indicated antibodies (**F**). A representative image of *n* = 2 two independent experiments is shown. ****p < 0.0001; **p < 0.01, *p < 0.05 and n.s., not significant, *p* > 0.05 by one-way ANOVA test.

JAK2 is a structurally flexible enzyme with large conformational changes existing between inactive and active states governed by phosphorylation of activation loop Tyr1007/Tyr1008. Lucet et al. revealed that several interactions within the kinase domain stabilize the well-ordered conformation of the activation loop. Among those, the interaction between the two antiparallel basic residues (β9/β6 and β10/β11) in the kinase domain and two arginine residues, Arg971 and Arg975, within the tip of the activation loop are important. In addition Lys999 residue establishes an intermolecular interaction with phosphorylated Tyr1008, which is important for stabilizing the activation loop conformation inside the kinase domain [22]. Previously, Lin et al. revealed the mechanism responsible for hyperphosphorylation of the Akt activation loop in the presence of Akt inhibitor GDC-0068. In their study, the authors demonstrated that when Akt is in an inhibited, conformationally restricted state, the phosphate group on Thr308 forms intramolecular interactions with Arg273 and Lys297 [23]. Based on this observation, we performed homology modeling of Akt with JAK2 and found that Arg273 and Lys297 of Akt are homologous to Arg976 and Lys 999 of JAK2. This made us question whether preventing R975 and K999 mediated interaction by alanine substitution leads to destabilization of the activation loop conformation as noticed in Akt. In line with our hypothesis, both JAK2-V617F + R975A and JAK2-V617F + K999A expressed in HEK293T cells exhibited reduced phosphorylation at Tyr1007/Tyr1008 of JAK2 compared to JAK2-V617F (Fig. 4A). Accordingly, less autophosphorylation of JAK2, and less STAT5 phosphorylation was found in JAK2-V617F + R975A and JAK2-V617F + K999A variants (Fig. 4A). Treatment of pervanadate rescued phosphorylation of JAK2 in V617F + R975A and V617F + K999A mutants, indicating that these residues play a critical role in the activation loop conformation of JAK2 (Fig. 4A) and destabilization of the activation loop conformation is prone to the action of phosphatases.

Surprisingly, the destabilization mutants JAK2-V617F + R975A and JAK2-V617F + K999A also led to a factor-independent growth of the Ba/F3 cells (Fig. 4B). Analysis of the destabilization mutants JAK2-V617F + R975A and JAK2-V617F + K999A with ruxolitinib treatment suggested that these mutants did not impair suppression of cell growth, suggesting that drug binding and activity of ruxolitinib is not affected by these mutants (Fig. 4C, E). Biochemical analysis of ruxolitinib treatment of JAK2-V617F + R975A mutants revealed increased phosphorylation of the activation loop at low concentrations (although reduced compared to JAK2-V617F) and a decrease in phosphorylation at higher concentrations (Fig. 4D), indicating that Tyr1007 interaction with Arg975 is required for the optimal protection of Tyr1007 phosphorylation from phosphatases. Similarly, treatment of JAK2-V617F + K999A mutants with ruxolitinib showed the absence of hyperphosphorylation at Tyr1007 and Tyr1008 residues (Fig. 4F), suggesting that phosphorylated Tyr1008 interaction with Lys999 is critical for the ruxolitinib-mediated stabilization of the Tyr1008 site. Taken together, these data indicate that ruxolitinib-induced paradoxical JAK2 hyperphosphorylation arises from protection of the activation loop tyrosines from phosphatases. In addition to the biochemical data, structural analysis of the JAK2 kinase domain revealed that the activation loop Tyr1007 is buried in the ruxolitinib bound state and substitution of Ala at Arg975 and Lys999 leads to exposure of the activation loop Tyr1007/1008 to the surface (Supplementary Fig. S7). Based on the study by Lucet et al., we introduced Ala at Lys1030 which is involved in interaction with phosphorylated Tyr1007. Interestingly, JAK2-V617F + K1030A showed less autophosphorylation of JAK2 and STAT5 (Supplementary Fig. S8A, B), and pervanadate treatment led to an increase in JAK2 and STAT5 phosphorylation (Supplementary Fig. S8A and B). Ruxolitinib treatment at lower concentrations showed less JAK2 hyperphosphorylation of JAK2-V617F + K1030A compared to JAK2-V617F, but at higher concentrations, led to an increase in activation loop Tyr1007/1008 phosphorylation (Supplementary Fig. S8C, D). Considering the greater effect of the interaction of Lys999 with phosphorylated Tyr1008 on ruxolitinib-induced hyperphosphorylation, our results suggest the greater importance of Tyr1008 interaction with residues to explain ruxolitinib-induced activation loop hyperphosphorylation.

#### Hyperphosphorylated JAK2 is hyperactive after ruxolitinib dissociation

**Fig. 5:**
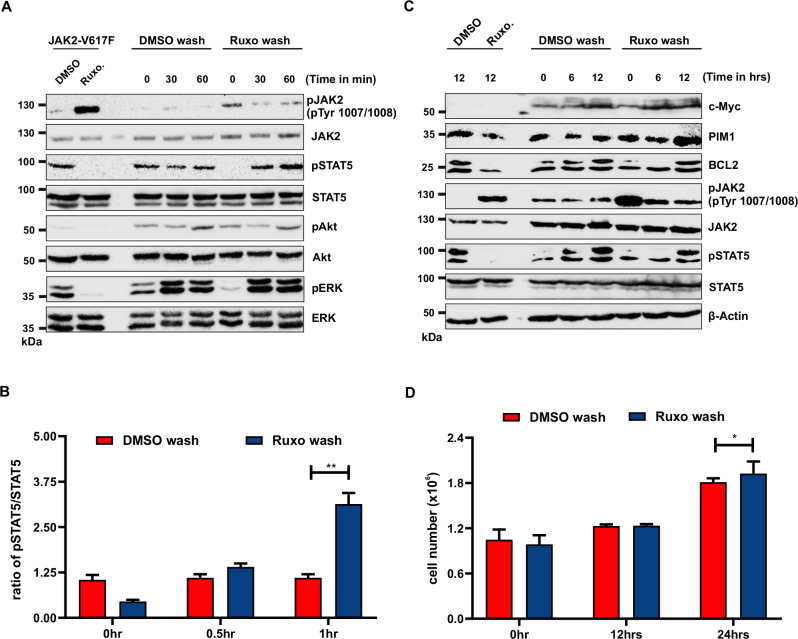
Hyper-phosphorylated JAK2 is hyperactive in in vivo. Ba/F cells expressing JAK2-V617F cells were treated for one hour with ruxolitinib and DMSO, and after one hour of pretreatment, ruxolitinib and DMSO is washed out from the cells with PBS (3 times) and incubated in the absence of inhibitor with indicated time points and lysates were measured for STAT5, AKT and ERK activity (**A**). Quantification of phsopho-STAT5 was measured with indicated time periods when lysates were prepared from ruxolitinib wash and DMSO wash (**B**). Similarly, ruxolitinib washed out and DMSO washed out samples were incubated with the indicated time period to measure the c-Myc, PIM1and Bcl-2 levels (**C**). Ba/F3 cells expressing the JAK2-V617F were treated with 1 μM ruxolitinib or DMSO for a time period of 45 min, and ruxolitinib and DMSO were washed out from cells and incubated for a period of 24-h and measured the cell proliferation using 3-(4,5-dimethylthiazol-2-yl)-5-(3-carboxymethoxyphenyl)-2-(4-sulfophenyl)-2h-tetrazolium (MTS)- based method. Data is shown as mean ± standard deviation (SD) (*n* = 3). OD—optical density (**D**). **p < 0.01, *p < 0.05 and n.s., not significant, *p* > 0.05 by Student’s *t* test.

Our data demonstrates that in the presence of Type I ATP-competitive inhibitors, JAK2 is hyperphosphorylated while STAT5 is suppressed (Fig. 1). However, when ruxolitinib was released from active JAK2 in Ba/F3 cells expressing JAK2-V617F, one hour after wash strong activation of STAT5 was observed compared to cells not exposed to ruxolitinib (Fig. 5A, B). However, no change in Akt activation and a modest increase in ERK1/2 were observed after one hour. (Fig. 5A). Consistent with rebound STAT5 activation, cells that were exposed to one-hour ruxolitinib treatment followed by washout showed increased expression of STAT5 target genes such as c-Myc and PIM1 but no change in Bcl-2 levels (Fig. 5C, supplementary Fig. S9). In order to measure the kinetics of ruxolitinib-induced paradoxical JAK2 activation loop Tyr1007/Tyr1008 phosphorylation, treatment with 1 μM ruxolitinib was extended for different time points. We found that 30 minutes of treatment was sufficient to induce activation loop Tyr1007/Tyr1008 phosphorylation (Supplementary Fig. S10). Consistent with this observation, cells exposed to ruxolitinib for one hour followed by ruxolitinib washout significantly increased cell proliferation compared to cells exposed to DMSO control (Fig. 5D). To investigate and reproduce our findings of rebound STAT5 activation and induction of PIM kinases in other models, we next performed ruxolitinib washout experiments on JAK2-V617F positive human HEL and SET-2 cell lines. Similar to our findings in Ba/F3 cells, both HEL and SET-2 cells displayed rebound activation of STAT5 and its target genes PIM1, PIM2 leading to hyperproliferation of cells after ruxolitinib washout compared to its control (Supplementary Fig. S11A–H).

#### Hyperphosphorylated JAK2 hyperactivates JAK2/STAT5 target gene expression

**Fig. 6:**
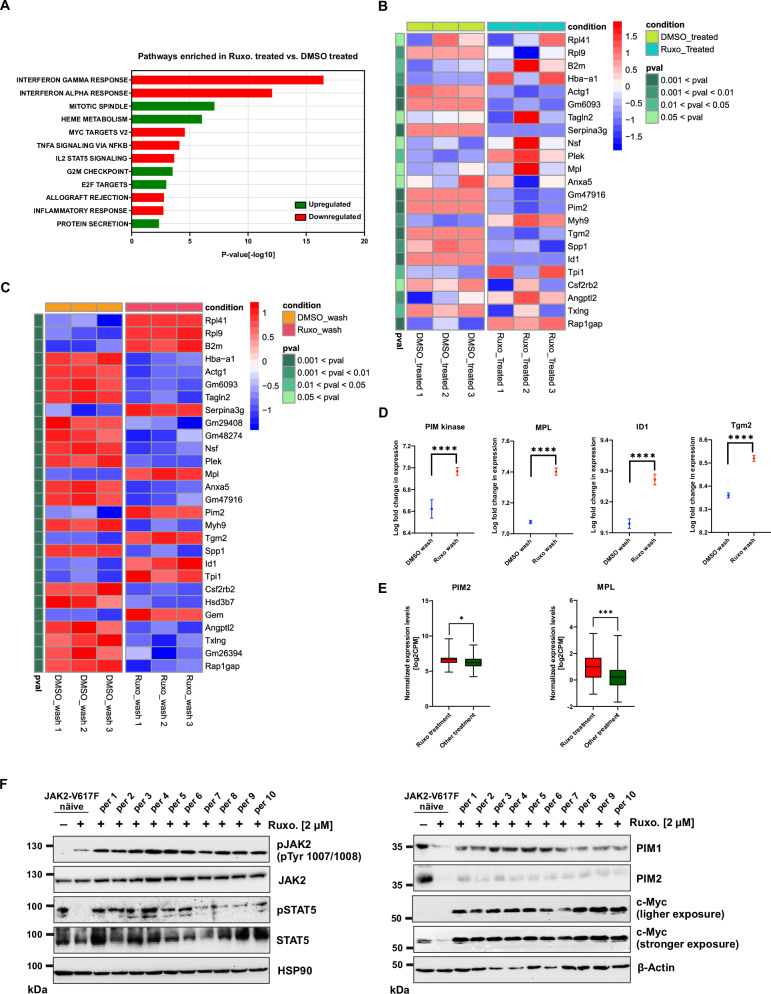
Ruxolitinib treated JAK2-V617 display hyperactivation of STAT5 target genes PIM, ID1 and MPL after ruxolitinib dissociation. Gene set enrichment analysis (GSEA) identifies Hallmark gene sets enriched in Ruxo-treated compared to DMSO-treated samples. The x-axis represents the -log10(*p* value), indicating the statistical significance of enrichment. The y-axis shows the names of the Hallmark gene sets. The color of each bar represents the direction of enrichment, with green indicating upregulation and red indicating downregulation of genes within the corresponding set (**A**). Heatmap depicting the log-CPM expression values (Z-score scaled) of selected genes across DMSO-treated and Ruxo-treated experimental conditions. The x-axis represents the experimental groups replicates, and the y-axis shows the individual genes. *P* value scale at the left provides statistical significance reference for interpreting the expression values (**B**). Heatmap depicting the log-CPM expression values (Z-score scaled) of selected genes across DMSO-wash and Ruxo-wash experimental conditions. The x-axis represents the experimental groups replicates, and the y-axis shows the individual genes. *P* value scale at the left provides statistical significance reference for interpreting the expression values (**C**). Quantification of representative values of *PIM2, ID1, MPL* and *TGM2* was shown between the DMSO wash and ruxolitinib wash samples (**D**). RNA-seq data of MPN patients treated with ruxolitinib (*n* = 36) and non-JAK inhibitor treatment (*n* = 126) was analyzed for *PIM2* and *MPL* transcripts levels (**E**). Immunoblot analysis of ruxolitinib persisters developed were subjected to indicated antibodies (**F**). A representative image of *n* = 2 two independent experiments is shown. ****p < 0.0001; ***p < 0.001, *p < 0.05 and n.s., not significant, *p* > 0.05 by Student’s *t* test.

To gain further insights into hyperactive JAK2 after ruxolitinib dissociation, we performed RNA-seq analysis in Ba/F3 cells expressing JAK2-V617F exposed to either DMSO or ruxolitinib at 1 μM for 6 hours (Fig. 6A, B). Ruxolitinib treatment led to an inhibition of canonical JAK2-signaling and STAT5 target genes such as *PIM* and *ID1*. In addition, we noticed a downregulation of *TGM2, Serpina 3 g, SPP1* and *ACTG1* (Fig. 6B). Next, we performed RNA-seq analysis on cells exposed to one-hour ruxolitinib, followed by ruxolitinib washout compared to DMSO washout cells. Ruxolitinib washout cells displayed a significant increase in PIM2, which is a crucial STAT5 target gene (Fig. 6C). Additionally, a significant upregulation of *ID1* was observed, which has been shown to be a JAK2-V617F target gene in MPNs [24] (Fig. 6C). Interestingly, *MPL* and *TGM2* were also significantly upregulated in ruxo-wash compared to DMSO-wash (Fig. 6C). Promoter analysis of *ID1* and *MP*L suggests that both genes have a consensus sequence TTCN_(2-4)_GAA of the GAS site by which STAT molecules bind as shown before [25] (Supplementary Fig. S12). To confirm that the transcriptional profile is specific for hyperactive JAK2 in the presence of ruxolitinib, we treated JAK2-V617F cells with a Type II JAK2 inhibitor (CHZ868), which does not induce JAK2 hyperphosphorylation (Supplementary Fig. S13A) and performed RNA-seq analysis in DMSO and CHZ868 treated,- and washout samples. As expected, CHZ868 treated samples showed inhibition of inflammatory and interleukin-related genes upon JAK2 inhibition (Supplementary Fig. S13B). However, CHZ868-wash failed to increase *PIM2, MPL, ID1*, and *TGM2* transcript levels in contrast to ruxo-wash samples (Supplementary Fig. S13C, D). In order to identify ruxolitinib mediated rebound activation of STAT5 target genes induction in MPN patients, we collected RNA-seq data of MPN patients treated with either ruxolitinib or other treatments (non-JAK inhibitor) from the MLL (Münchner Leukämielabor GmbH). RNA-seq analysis revealed significant upregulation of *PIM2* and *MPL* genes in patients treated with ruxolitinib compared to the non-JAK inhibitor treatment cohort (Fig. 6D). In line with our data, a previous study by Chen et al. showed a significant increase in *PIM2* levels after developing resistance to ruxolitinib in MPN patient [26]. Since PIM kinases were upregulated on the protein level (Fig. 5C) and transcript level (Fig. 6C) in ruxolitinib-treated samples, we generated Ba/F3 cell lines expressing JAK2-V617F, resistant to ruxolitinib at 2 μM and 4 μM. Co-treatment with the PIM inhibitor TP-3654 at 500 nM drastically reduced the number of ruxolitinib-resistant clones at 2 μM and 4 μM concentrations (Supplementary Fig. S14). These results demonstrate that ruxolitinib-dependent upregulation of PIM kinases contributes to ruxolitinib resistance and suggests that co-treatment of ruxolitinib with PIM kinase inhibitors prevents the development of ruxolitinib resistance. Upregulation of PIM1 and to some extent also PIM2 was confirmed in ruxolitinib-resistant sublines (Fig. 6E). Interestingly, ruxolitinib treatment in naïve cells leads to downregulation of PIM kinases (Fig. 6E). Of note, all resistant sublines that displayed strong c-Myc and PIM1 expression seemed to correlate with STAT5 phosphorylation and inversely correlated to c-Myc expression. Consistent with this, analysis of RNA-seq data from ruxolitinib and fedratinib persistent HEL clones deposited by Kong et al. [27] displayed significant upregulation of *PIM* kinases and *ID1* compared to parental cells (Supplementary Fig. S15A, B) suggesting that rebound activation of PIM kinases play a major role in type I inhibitor persistence due to non-permanent occupation of JAK2 with these inhibitors.

#### PIM kinases play an essential role in ruxolitinib-induced hyperproliferation and resistance towards ruxolitinib

**Fig. 7:**
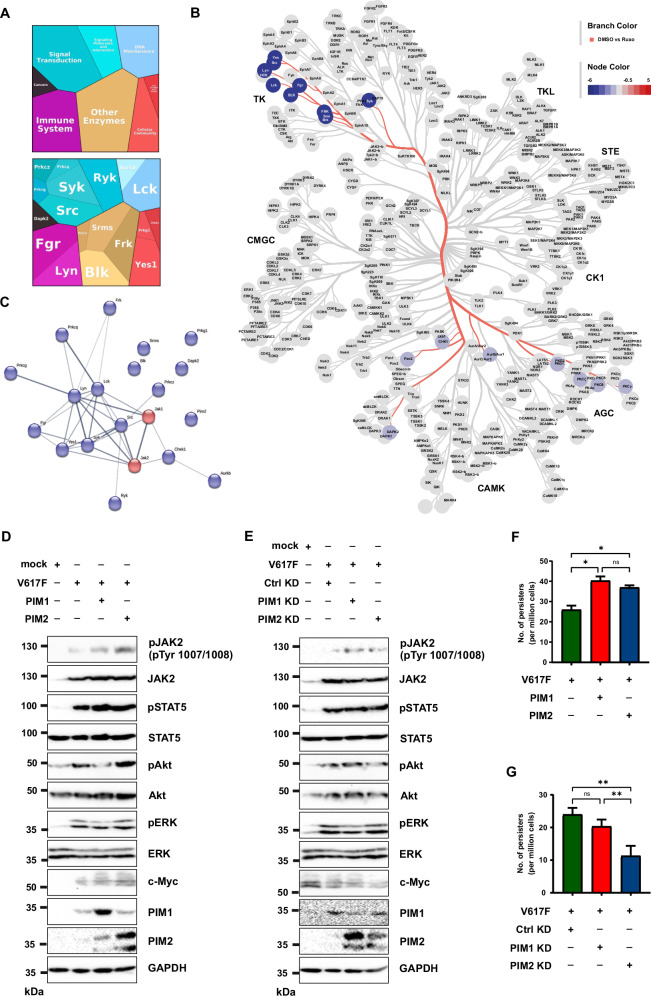
PIM kinases play an indispensable role in ruxolitinib-induced hyperproliferation and persistence. The proteomap illustrates the most altered kinases, represented as polygon-shaped tiles, with their mean kinase statistics. Proteins within the same category are color-coded similarly and positioned adjacently to form larger regions. In this case, the activity of all displayed kinases is reduced compared to the control (DMSO) due to the effective inhibition of JAK kinases by ruxolitinib. The size of each polygon is proportional to the magnitude of change (**A**). Tyrosine and serine/threonine kinases affected by ruxolitinib inhibition of JAK1/2 in the phylogenetic kinome tree (**B**). String network (medium confidence of 0.400) for kinases influenced by the ruxolitinib inhibition of JAK1/2 (JAK1/2 shown in red, blue = reduction of kinase activity), represented by the mean kinase statistic (**C**). PIM1 and PIM2 were ectopically expressed in Ba/F3 JAK2-V617F cells, and lysates were subjected to western blotting with the indicated antibodies (**D**). PIM1 and PIM2 were stably knockdown in Ba/F3 JAK2-V617F cells, and lysates were subjected to western blotting with the indicated antibodies (**E**). Single clones of Ba/F3 cells expressing JAK2-V617F with PIM1 and PIM2 kinases grown in 96-well plates in the presence of 4 μM ruxolitinib were counted. The number of resistant clones per million cells input is shown (**F**). Single clones of Ba/F3 JAK2-V617F with PIM1 and PIM2 knockdown cells in 96-well plates with 4 μM ruxolitinib were counted. The number of resistant clones per million cells input is shown (**G**). **p < 0.01, *p < 0.05 and n.s., not significant, *p* > 0.05 by Student’s *t* test.

**Fig. 8:**
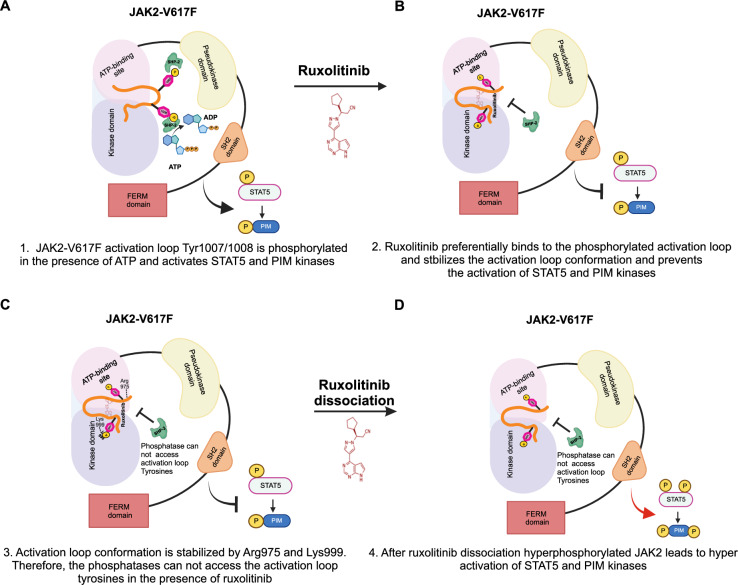
Model of paradoxical activation of JAK2-V617F in the presence of ruxolitinib. In the presence of ATP, activation loop Tyr1007/Tyr1008 of JAK2-V617F is phosphorylated, which leads to activation of STAT5 and PIM-kinases (**A**). Ruxolitinib binds to the kinase domain of JAK2, when activation loop Tyr1007/Tyr1008 are fully phosphorylated. This binding stabilizes the activation loop conformation inside the kinase domain of JAK2. Thus, the phosphatases cannot access the phosphorylated tyrosine residues of activation loop, however JAK2 in this conformation exists in an inhibited state (**B**). Arg975 and Lys999 residues help in the stabilization of the activation loop conformation inside the kinase domain (**C**). When hyperphosphorylated JAK2 is dissociated from ruxolitinib, it leads to hyperactivation of STAT5 and PIM-kinases (**D**).

To gain a deeper insight into the ruxolitinib-induced paradoxical increase of activation loop tyrosines and examine kinase inhibition by ruxolitinib, we performed kinase activity profiling using PamChip®4. PamChip analysis results showed that kinases that are involved in signal transduction, immune system regulation, cancer pathways, cell growth and cell death were significantly affected by ruxolitinib treatment (Fig. 7A). String analysis illustrates influenced kinases and their crosslink which were mainly affected by the targeted inhibition of JAK1 and JAK2 after ruxolitinib treatment. Among the protein tyrosine kinases (PTKs), the SRC family kinases were most strongly inhibited. Regarding the serine/threonine kinases, PIM2, together with CHK1 and DAPK2, were inhibited with ruxolitinib treatment (Fig. 7A–C). Ruxolitinib recovery after 15 min showed an increase in the activity of kinases that are involved in signaling pathways, such as JAK-STAT, chemokine, Ras and Erbb compared to ruxolitinib treatment. (Supplementary Figure S16A–C). Since ruxolitinib treated MPN patients displayed significant induction of PIM2 levels, we suspected that PIM kinases might be involved in ruxolitinib-induced hyperproliferation and persistence. For this purpose, we ectopically expressed PIM1 and PIM2 in JAK2-V617F expressing Ba/F3 cells and found that PIM kinases induced c-Myc and Akt, as shown before [28, 29] (Fig. 7D). PIM1 and PIM2 overexpressing JAK2-V617F cells exhibited hyperproliferation after ruxolitinib washout compared to DMSO washout (Supplementary Fig. S16D). Consistent with the hyperproliferation, PIM1 and PIM2 expressing cells displayed a greater number of ruxolitinib persisters at 4 μM concentration (Fig. 7F). In contrast to these data, knockdown of PIM1 and PIM2 led to a decrease in c-myc and Akt activation (Fig. 7E), and inhibition of ruxolitinib-induced hyperproliferation (Supplementary Fig. S16E). PIM2 knockdown displayed fewer ruxolitinib persisters at 4 μM concentration (Fig. 7G). Collectively, our study uncovers the significance of paradoxical JAK2-V617F activation loop hyperphosphorylation in the presence of type I JAK inhibitors. In the presence of ATP, JAK2-V617F leads to the phosphorylation of the activation loop Tyr1007/Tyr1008 activating STAT5 and PIM kinases (Fig. 8A). Ruxolitinib preferentially binds when the activation loop Tyr1007/Tyr1008 are fully phosphorylated (Fig. 8B). The conformation of the activation loop within the kinase domain is stabilized by ruxolitinib binding and supported by interactions of Tyr1007/Tyr1008 to Arg976 and Lys999. This protects the activation loop tyrosines from phosphatases, which leaves Tyr1007/Tyr1008 in a state of paradoxical hyperphosphorylation, but JAK2 in an inactive state (Fig. 8C). After ruxolitinib release, hyperphosphorylation of JAK2 Tyr1007/Tyr1008 leads to a rebound hyperactivation of STAT5 and PIM kinases (Fig. 8D). This mechanism may also contribute to persistence of MPN in patients receiving ruxolitinib who do not acquire mutations in the kinase domain of JAK2. Our results demonstrate that PIM kinases are a critical downstream mediator of hyperactivated JAK2. As co-treatment with a PIM kinase inhibitor prevented ruxolitinib resistance in vitro, our data supports the rational of using PIM inhibitors as a combination treatment with JAK inhibitors in JAK mutated MPN.

## Discussion

JAK2 small molecular inhibitors such as ruxolitinib, fedratinib, momelotinib and lestaurtinib display remarkable clinical activity in clinical trials in PV and MF patients. Ruxolitinib has been approved for the treatment of primary and secondary myelofibrosis. However, responses are of limited duration, and the mechanism of ruxolitinib action on constitutively activated JAK2 is not entirely understood. Previous studies demonstrated that ruxolitinib treatment leads to a paradoxical increase in phosphorylation of activation loop tyrosines [19, 30]. This paradoxical hyperphosphorylation occurs subsequent to the binding of type I inhibitors to the active conformation [14], but the precise mechanism of increase in tyrosine phosphorylation of the activation loop is still to be elucidated. Tvorogov et al. demonstrated that accumulation of activation loop tyrosine phosphorylation is responsible for the type I JAK2 inhibitor withdrawal syndrome. However, the effector molecules responsible for this mechanism were not described. In this study, we found that ruxolitinib binding stabilizes the activation loop conformation inside the kinase domain, protecting the activation loop tyrosines from phosphatases. Our study also identified the residues Arg975 and Lys999 which had not been shown before to be part of the regulation of the activation loop conformation for protection from access to phosphatases. This step helps the kinase until it catalyzes the second step of the reaction (substrate phosphorylation or autophosphorylation of other tyrosine residues within JAK2) by rendering JAK2 in an active state.

In this study, we observed that both kinase-dead JAK2 (V617F + K889R) and the ruxolitinib-resistant JAK2 (V617F + L983F) mutants did not exhibit paradoxical JAK2 hyperphosphorylation in the presence of the inhibitor, suggesting that ruxolitinib induces JAK2 hyperphosphorylation by an intrinsic mechanism. Our study also ruled out that ruxolitinib-mediated hyperphosphorylation of activation loop tyrosines was not due to blocking the enzymatic activity of the pseudokinase domain (Fig. 2). Using non-denaturing native immunoprecipitation, we demonstrated that in the presence of ATP-competitive inhibitors activation loop conformation is stabilized inside the kinase domain. Thus, the activation loop conformation plays a major role in allowing access of phosphatases to the activation loop, and the stabilization of the activation loop conformation inside the kinase domain might protect phosphatase-mediated dephosphorylation of the activation loop tyrosines. This method can be used to examine changes in the activation loop conformations in the presence of inhibitors, which demonstrates paradoxical kinase activation in the field of drug discovery. Our results align with previous reports on Akt, where GDC-0068 binding led to stabilization of Akt activation loop and prevented the access of phospho-specific antibodies (Thr308 and Ser473) to Akt [23].

Structural interrogation of the JAK2 kinase domain bound to a pan-JAK inhibitor suggested that a number of lysine residues stabilize the position of pTyr1007 and pTyr1008. Among those are pTyr1007 with Lys1005, Lys1009, Lys1030, and pTyr1008 with Lys999 [22]. However, no biochemical data is available so far to demonstrate the significance of these residues in stabilizing the activation loop when Tyr1007/Tyr1008 are fully phosphorylated. In this study, we identified two crucial residues, Arg975 and Lys999, playing a major role in stabilization of the activation loop conformation. Our biochemical results demonstrate that disruption of Arg975 and Lys999 interactions mediates activation loop destabilization and enhances the recruitment of phosphatases to the activation loop (Fig. 5). Likewise, inhibition of phosphatase enzymatic activity in these mutants rescued the phosphorylation of JAK2 suggesting that these two crucial residues mediate JAK2 activation loop conformation. It was also noticed that ruxolitinib treatment of JAK2-V617F + R975A and JAK2-V617F + K999A mutants abolished the inhibitor-induced JAK2 hyperphosphorylation compared to JAK2-V617F, demonstrating that paradoxical JAK2 hyperphosphorylation is due to the protection of activation loop tyrosines from phosphatases in the presence of inhibitor.

Finally, our results conclude that dissociation of ruxolitinib leaves JAK2 in a rebound hyperactivation of downstream signaling molecules. Koppikar et al. found ruxolitinib persistence linked to the heterodimerization of active JAK2 with other JAK family kinases [19]. This is consistent with our findings as we noticed activation of JAK3 after ruxolitinib recovery by PamGene analysis and rebound activation of STAT5 following ruxolitinib dissociation from active JAK2, which in turn induced PIM kinases, which according to our findings mediate hyperproliferation induced by rebound JAK activation. Importantly, this mechanism is not specific for ruxolitinib-bound JAK2-V617F. Due to the non-permanent occupation of JAK2 molecules with ruxolitinib, intermittent rebound activation of JAK2 downstream signaling might contribute to disease persistence and the limited anti-clonal effect observed in MPN patients treated with JAK1/2 inhibitors. In addition, responses to ruxolitinib in MF occur in JAK2-V617F and JAK2-WT patients. Since we observed ruxolitinib-induced paradoxical hyperphosphorylation both in JAK2-WT as well as JAK2-V617F and the homologous JAK1-V658F, we assume that unmutated and mutated JAK kinases contribute to downstream activation mediated by activation loop hyperphosphorylation in intermittent drug-unbound states in patients treated with type I JAK inhibitors including ruxolitinib, fedratinib, momelotinib and lestaurtinib.

Upon ruxolitinib dissociation, we found increase of PIM-kinase transcriptional activation and protein expression, suggesting that PIM kinases contribute to ruxolitinib resistance and persistence of MPN in patients receiving ruxolitinib treatment. Accordingly, previous studies demonstrated the synergistic effect of ruxolitinib and PIM kinase inhibitors in in vitro and in vivo models [31, 32]. Our study provides new mechanistic insights that might contribute novel treatment strategies for MPN patients and strongly rationalize the therapeutic efficacy of ruxolitinib in combination with PIM kinase inhibitors as shown by Dutta et al. [32] and the ongoing study investigating TP-3654 in patients with myelofibrosis who failed previous JAK inhibitor treatment (NCT04176198). In addition to PIM kinases, we observed induction of *ID1* transcription in ruxolitinib treated cells. ID1 was identified as a novel target of JAK2-STAT5 signaling in erythroid cells. STAT5 binds and transactivates a downstream enhancer of ID1, and ID1 expression levels correlate with the JAK2-V617F mutation in both retrovirally transfected fetal liver cells and PV patients [24]. Thus, inhibition of ID1 signaling in JAK2-V617F mediated MPN might contribute to a positive clinical outcome, as ID1 maintains the self-renewal capacity of myeloid progenitor cells [33]. RNA-seq data from ruxolitinib washout samples demonstrated *MPL* upregulation, specifically in ruxolitinib treated samples. Previously, it has been demonstrated that MPL overexpression leads to ruxolitinib resistance in MPN with calreticulin frame-shift mutations [34]. In line with this, our RNA-seq data suggests that MPL could be a potential downstream target of STAT5, and targeting MPL signaling axis might be beneficial in MPN patients refractory to ruxolitinib. In contrast to STAT5 target genes such as *PIM, ID1* and *MPL*, we noticed significant induction of *TGM2* transcript levels in ruxolitinib treated samples. The functional role of TGM2 in MPNs is not understood. However, it was previously demonstrated that TGM2 is upregulated in T-cell lymphoblastic lymphoma and the IL-6/JAK/STAT3 signaling axis was activated downstream of TGM2 [35]. In addition, TGM2 levels were upregulated in AML and TGM2 was identified as an independent prognostic factor in non-small cell lung cancer [36, 37]. Nevertheless, the significance of TGM2 in JAK2-V617F mediated MPN warrants further investigation. Thus, development of new generation JAK2 inhibitors, such as allosteric inhibitors which block the activity of JAK2, might be more beneficial for the treatment of JAK2-mediated MPNs.

Together, this work elucidates the mechanism and demonstrates the clinical significance of ruxolitinib-mediated paradoxical hyperphosphorylation of JAK2-V617F and suggests that PIM kinase inhibitors are rational candidates for combination treatment with type I JAK inhibitors in MPN.

## Supplementary information

Supplementary Files

PamGene _DMSO vs Ruxo_PTK

PamGene_DMSO vs Ruxo_STK

PamGene_Ruxo vs Ruxo wash_PTK

PamGene_Ruxo vs Ruxo wash_STK

MPN patients Ruxo vs non-JAK inhibitor treatment

## Data Availability

RNA-sequencing data generated in this study is available at NCBI: GSE284481. PamGene analysis and MPN patients’ RNA-seq data for target genes PIM1, PIM2, ID1, MPL, TGM2, GEM and Serpina are available in the supplementary file.
