# Supplementary material for: Ruxolitinib mediated paradoxical JAK2 hyperphosphorylation is due to the protection of activation loop tyrosines from phosphatases
Source: Leukemia. 2025 Apr 23;39(7):1678–91. doi: 10.1038/s41375-025-02594-7 (PMC12208895; doi:10.1038/s41375-025-02594-7)
Supplement: Supplementary file 1 — Supplementary Files [file 41375_2025_2594_MOESM1_ESM.docx]

**Ruxolitinib mediated paradox JAK2 hyperphosphorylation is due to the protection of activation loop phosphotyrosines from phosphatases**

**Supplementary Materials and Methods:**

**Cell culture and DNA constructs**

Ba/F3 cells were obtained from the German Resource Centre for Biological Material (DSMZ) in 2005 and authenticated by DNA typing, species PCR and immunophenotyping. Cells were passaged for less than 6 months and were maintained in the presence of 2ng/ml interleukin-3 (R&D, Wiesbaden, Germany). Ba/F3 cells were transfected by retroviral gene transfer and transformed upon withdrawal of interleukin-3. Ba/F3 cells were maintained in RPMI 1640 medium containing 10% fetal calf serum in the presence of murine IL-3. HEK293T cells were maintained in Dulbecco modified Eagle medium (Gibco, Thermo Fisher, USA) containing 10% fetal calf serum. Human WT JAK2 and V617FJAK2 were cloned into the EcoRI site of the MigRI retroviral vector expressing the enhanced yellow fluorescent protein (eGFP) as described previously^1^. JAK2 mutations S523A, K581R, F983F, R975A and K999A were introduced in MSCV-EYFP-V617FJAK2 using the QuickChange mutagenesis kit (Stratgene, Amsterdam, The Netherlands). JAK1 WT and JAK1-V658F was cloned into EcoRI site of MSCV-Puro retroviral vector. The L1010F mutation in JAK1 was inserted with the help of the QuickChange mutagenesis kit (Stratgene, Amsterdam, The Netherlands). PIM1 and PIM2 over expression and knockdown studies in Ba/F3 cells were performed as described previously^2^.

**Western blot and co-immunoprecipitations**

Ba/F3 cells were cultured for 2.5 hours in the presence of the respective inhibitor at the indicated concentrations. Cell lysis, sodium dodecyl sulfate–polyacrylamide gel electrophoresis (SDS-PAGE), and immunoblotting were done as described previously^3^. Bands were visualized using the enhanced chemiluminescence (ECL) system (Amersham, Braunschweig, Germany). For co-immunoprecipitation studies, 20x10^6^ cells were harvested after indicated treatment, washed with PBS, and collected in RIPA lysis buffer containing protease inhibitor cocktail (Roche). Lysates were pre-cleared with either protein A or protein G beads for 30 minutes, followed by incubation with primary antibody overnight. The immunoprecipitated fraction was washed three times with lysis buffer and dissolved in SDS-loading buffer, and equal amounts were loaded on SDS-PAGE gels. The beads were then washed and bound fractions were subjected to SDS-PAGE and transferred to polyvinylidene difluoride (PVDF) membranes. Bound JAK2 protein was visualized by western blotting using anti-JAK2 antibody.

**Denaturing and non-denaturing immunoprecipitations**

For denaturing immunoprecipitation Ba/F3 cells expressing the various JAK2 mutants were treated for three hours with the indicated inhibitors. Cells were harvested with lysis buffer (20mM Tris (PH 7.4), 150mM NaCl, 1% nonidet P-40, 2mM PMSF, 2mM sodium orthovanadate and cleared by centrifugation. Immunoprecipitations were carried out on cell lysates by pre-clearing the lysates with protein A agarose beads and followed by incubating with antibody of interest in the cold room at 4^0^C overnight. Flag IPs were performed with agarose conjugated anti-flag beads (Sigma), whereas JAK2 IPs were carried out using the anti JAK2 conjugated agarose beads (Upstate). pJAK2 IPs were performed with rabbit polyclonal antibody against the phospho Tyr1007/1008 (Santa Cruz Biotechnology 21870-R) overnight. The immunoprecipitated fraction was isolated by incubating with protein A agarose beads, and immunoprecipitants were washed three times with lysis buffer before eluting with Laemelli buffer. For non-denaturing immunoprecipitation Ba/F3 cells expressing the various JAK2 mutants were treated for three hours with the indicated inhibitors. Cells were harvested with a buffer containing 20mM Tris (PH 7.4), 100mM NaCl, 2mM PMSF, 2mM sodium orthovanadate, and the cells were sonicated using a Bioruptor (power: H position, sonication cycles: 30 sec on/30 sec off, total sonication cycles: 5-15 and temp: 4^0^). After sonication, clear lysates were obtained by centrifugation and supernatant was incubated with indicated antibodies as mentioned in denaturing immunoprecipitation.

**RNA-sequencing and analysis**

Ba/F3 cells expressing JAK2-V617F were treated with 1μM ruxolitinib, CHZ868 and DMSO for 6 hours, and RNA was isolated from these cells. RNA-seq analysis was performed using BMK GmbH (Münster, Germany). For ruxolitinib washout RNA-seq analysis, Ba/F3 cells expressing JAK2-V617F were treated with 1μM ruxolitinib for 1 hour, after which ruxolitinib was washed out by repeated (3x) PBS washing and incubated without ruxolitinib for 6 hours. RNA was isolated using the Trizol method and samples were stored at -80°C. RNA-seq analysis was performed by BMK GmbH (Münster, Germany). Samples were sequenced using the Illumina NovaSeq 6000 S4 flow cell, and raw sequencing reads were pseudo-aligned to the GRCm38 mm10 genome assembly using ***kallisto***^4^. Transcript read counts were aggregated to Ensembl Gene IDs using the R package ***biomaRt*** for further analysis^5, 6^. Raw transcript read counts were converted to CPM and log-CPM values using the ***cpm*** function in ***edgeR*** package ^7, 8^ ^9^ ^10^, and stored in a DGEList object. Genes with low expression were removed, retaining those with a CPM ≥ 1.34 in at least three samples. Gene expression was then normalized using the Trimmed Mean of M-values (TMM) method in ***edgeR***, correcting for biases in sequencing depth or library size. For differential gene expression analysis, heteroscedasticity was addressed using the **voom** function in ***limma***, which applied precision weights to log-CPM values^11^. A linear model was fitted to each gene’s expression using **lmFit** in ***limma***, and empirical Bayes moderation was performed to obtain more precise estimates of gene-wise variability. Gene set enrichment analysis was performed using ***gage***^11^ and ***msigdbr***^12^ to identify gene sets that were significantly enriched between cell groups. Statistically significant gene sets were determined using a q-value threshold of 0.05.

**Kinase activity profiling**

The kinase activity of Ba/F3 cell lysates was quantified using the PamChip®4 platform (PamGene International B.V.,’s-Hertogenbosch, The Netherlands) by measuring the phosphorylation of peptide representations of kinase targets or substrates immobilized on PamChip® microarrays. Ba/F3 cells expressing JAK2-V617F were treated with 1 μM ruxolitinib or DMSO for 3 hours, followed by protein isolation according to the manufacturer's protocol for cell lysate preparation. For ruxolitinib washout analysis, cells were treated with 1 μM ruxolitinib, followed by PBS washes to remove the inhibitor. Kinase activity was also profiled at 15 minutes post-washout. Lysates were stored at -80°C until further analysis. Protein tyrosine kinase (PTK) and serine/threonine kinase (STK) activities were analyzed as previously described^13^. Differences concerning the kinase activity profile were validated by comparing the phosphorylation state of the ruxolitinib-treated Ba/F3 cells to the DMSO cultured cells, or the ruxo-wash out (15minutes) to the ruxolitinib treated cells. The mean kinase statistic was used for further analysis: The proteome was visualized using proteomaps database^14^, Kinome Trees were created using the Coral^15^ or STRING database^16^.

**Structural analysis of the kinase domain (JH1) of the JAK family**

The structures of JAK2 JH1 domain in complex with CMP-6 (PDB-2B7A), JAK3 JH1 in complex with CMP-6 (PDB-3LXL), JAK2 JH1 domain in complex with ruxolitinib (PDB-6VGL) and JAK3 JH1 in complex with Tofacitinib (PDB-3LXK) were obtained from the RCSB PDB database (<https://www.rcsb.org/>). The structures of the kinase domains of JAK2-R975A and JAK2-K999A were generated by providing the respective sequences into the Alphafold3 server (<https://alphafoldserver.com/>). The structures were visualized using PyMOL by Schrödinger (<https://www.pymol.org/>).

**Figure S1. JAK2-specific ATP-competitive inhibitors leads to paradoxical JAK2 hyperphosphorylation but not JAK3:** Ba/F3 cells expressing the JAK2-V617F were treated with the indicated concentration of momelotinib (A) and BMS-911543 (B) for 3 hours. Lysates were prepared and subjected to western blotting for the pJAK2, JAK2, pSTAT5, STAT5 and HSP90. HEL cells (JAK2-V617F^+^) were treated for 3 hours with ruxolitinib and lysates were analyzed for activation of JAK2 and STAT5 (C). Ba/F3 cells expressing the JAK3-WT were treated with indicated concentrations of tofacitinib in the presence of IL-2 and lysates were subjected to western blotting for the pJAK3, JAK3, pSTAT3, STAT3 and GAPDH (D).

**Figure S2. Activation loop conformation of JAK2 differs from JAK3 in inhibitor-bound state:** Molecular surface representation of JAK2 kinase domain with CMP-6 (pan-JAK inhibitor). Activation loop (994-1023) of JAK2 is colored in green. Activation loop Tyr1007 is colored in orange and Tyr1008 is colored in blue. Both the activation loop Tyr1007 and Tyr1008 were buried inside the kinase domain (A). Molecular surface representation of JAK3 kinase domain with CMP6. Activation loop (967-996) of JAK3 is colored in green. Activation loop Tyr980 is colored in orange and Tyr981 is colored in blue. Tyr 980 and 981 both were exposed in CMP-6 bound conformation. (B). Molecular surface representation of JAK2 kinase domain with ruxolitinib. Activation loop Tyr1008 is exposed and Tyr1007 was not visible in surface representation indicating that Tyr1007 is buried inside the kinase domain (C). Molecular surface representation of JAK3 kinase domain with tofacitinib. Both the activation loop tyrosines were exposed in tofacitinib bound conformation (D). Superimposition of JAK2 kinase domain with JAK3 kinase domain. JAK2 is colored in green, JAK3 is colored in magenta. Differences in the activation loop conformation is highlighted with an arrow (E).

**Figure S3. Pseudokinase domain kinase activity is not involved in paradox hyperphosphorylation of JAK2:** Proliferation of parental Ba/F3 cells and Ba/F3 cells expressing JAK2-V617F, JAK2-V617F+S523A and JAK2-V617F+K581R in the absence of IL-3 was quantified by the relative optical density (OD) with the indicated time period using a 3-(4,5-dimethylthiazol-2-yl)-5-(3-carboxymethoxyphenyl)-2-(4-sulfophenyl)-2h-tetrazolium (MTS)- based assay (A). HEK293 cells expressing mock, JAK2-WT, JAK2-V617F, JAK2-V617F+S523A and JAK2-V617F+K581R were serum starved for 12 hours, and lysates were subjected to western blotting with the indicated antibodies (B). Ba/F3 cells expressing the JAK2-V617F, JAK2-V617F+S523A and JAK2-V617F+K581R were treated with the indicated concentration of ruxolitinib and proliferation was measured using MTS after incubation for 48hrs in the presence of increasing concentrations of the inhibitor ruxolitinib (C and E). Data is shown as mean ± standard deviation (SD) (n=3). OD – optical density. Immunoblot analysis of Ba/F3 cells expressing JAK2 mutants cultured with indicated concentrations of ruxolitinib for 3 hours and lysates were subjected to indicated antibodies (D and F). A representative image of n=2 two independent experiments is shown. ****p<0.0001; **p<0.01, and *p<0.05 by Student’s t test.

**Figure S4. Ruxolitinib treatment does not prevent the association of phosphatases with JAK2:** Ba/F3 cells expressing JAK2-V617F were treated with the 1μM ruxolitinib for 3 hours and 1μM pervanadate for 30 minutes. Lysates were prepared for JAK2 immunoprecipitation (IP). JAK2 immunoprecipitation (IP) fractions were subjected to immunoblotting to verify SHP-2 interaction (A). Whole cell lysate (WCL) was probed with the indicated antibodies to show the expression levels of JAK2 and SHP-2 (B).

**Figure S5. Upstream kinases are not involved in ruxolitinib-induced paradox JAK2 hyperphosphorylation:**  JAK2-V617F and JAK2-V617F+K889R were treated with vehicle or ruxolitinib with indicated concentrations for 3 hours, and lysates were subjected to western blotting for indicated antibodies.

**Figure S6: Inhibition of phosphatase’s action followed by ruxolitinib treatment prevents the paradox JAK2 hyperphosphorylation:** Ba/F3 cells expressing JAK2-V617F cells are co-treated with phosphatase inhibitor (vanadate) and JAK2 inhibitor (ruxolitinib) for a period of 1 hour and lysates were subjected to the indicated antibodies (left panel). Similarly, vanadate was washed out from the cells, and lysates were subjected to pJAK2 to measure the hyperphosphorylation of activation loop Tyr1007/1008 (right side panel).

**Figure S7. JAK2-R975A and K999A leads to destabilization of the activation loop conformation and exposes the activation loop to the surface:** Molecular surface representation of JAK2 kinase domain with ruxolitinib. Tyr1008 is colored in blue and Tyr1007 is colored in orange. Only blue can be visualized indicates that the activation loop Tyr1008 is exposed while the Tyr1007 is buried inside the kinase domain (A). Molecular surface representation of the JAK2 kinase domain with CMP-6. Activation loop Tyr1007 is colored in orange and Tyr1008 is colored in blue. Absence of both colors indicates that Tyr1007/1008 are buried inside the kinase domain (B). R975K and K999A structures were generated using Alphafold3 and molecular surface representations were visualized using PyMol. Tyr1007 is colored in orange and Tyr1008 is colored in blue. Both structures displayed exposure of Tyr1007/1008 (C-D). Differences in the activation loop of each structure were highlighted with dotted circle.

**Figure S8. Prevention of interaction of Lys1030 with phospho-Tyr1007 leads to destabilization of the activation loop:**  HEK293T cells expressing the JAK2-V617F and JAK2-V617F+K1030A were serum starved and treated with or without 1μM pervanadate. Lysates were subjected to indicated antibodies (A). Quantitative ratio of pSTAT5 versus STAT5, pJAK2 versus JAK2 of JAK2-V617F and JAK2-V617F+K10130A expressing cells in presence and absence of pervanadate were quantified using the ImageJ software (B). Immunoblot analysis of HEK293 cells expressing JAK2 mutants cultured with indicated concentrations of ruxolitinib for 3 hours, and lysates were subjected to indicated antibodies (C). Quantitative ratio of pJAK2 versus JAK2 of JAK2-V617F and JAK2-V617F+K10130A expressing cells treated with ruxolitinib were quantified using the ImageJ software (D). **p<0.01, *p<0.05, n.s., not significant, p>0.05 by Student’s t test.

**Figure S9. PIM1 is upregulated after ruxolitinib dissociated from hyperactive JAK2:** Ba/F3 cells expressing the JAK2-V617F were treated with 1μM ruxolitinib or DMSO for one hour, and ruxolitinib and DMSO were subsequently washed out from cells. These cells were then incubated for the indicated time period and lysates were subjected to c-Myc and PIM1 antibodies. Western blot images were quantified with the ImageJ software and relative protein levels were measured relative to ß-actin (A and B). ***p<0.001, n.s., not significant, p>0.05 by Student’s t test.

**Figure S10. Turnover time of ruxolitinib-induced JAK2 hyperphosphorylation of the activation loop Tyr1007/1008:** Ba/F3 cells expressing JAK2-V617F were treated with 1μM ruxolitinib for different time periods as indicated. Lysates were subjected to pJAK2 to detect the time period required to show the activation loop hyperphosphorylation.

**Figure S11. Hyperphosphorylated JAK2 is hyperactive *in vivo*:** HEL cells (JAK2-V617F^+^) were treated for one hour with ruxolitinib and DMSO. After one hour of pretreatment, ruxolitinib and DMSO was washed out from the cells with PBS (3 times) and incubated in the absence of the inhibitor for the indicated time points. Lysates were measured for STAT5, Akt and ERK activity (A). Quantification of pSTAT5 was measured with indicated time periods when lysates were prepared from ruxolitinib wash and DMSO wash (B). Similarly, ruxolitinib washout and DMSO washout samples were incubated for the indicated time period to measure c-Myc, PIM1, PIM2 and ID1 levels (C). HEL cells were treated with 1μM ruxolitinib or DMSO for a time period of 45 minutes, ruxolitinib and DMSO were washed out from cells and incubated for a period of 24 hours. Cell proliferation was measured using 3-(4,5-dimethylthiazol-2-yl)-5-(3-carboxymethoxyphenyl)-2-(4-sulfophenyl)-2h-tetrazolium (MTS)- based method. Data is shown as mean ± standard deviation (SD) (n=3). OD – optical density (D).

SET-2 cells (JAK2-V617F^+^) were treated for one hour with ruxolitinib and DMSO. After one hour of pretreatment, ruxolitinib and DMSO was washed out from the cells with PBS (3 times) and incubated in the absence of the inhibitor for the indicated time points. Lysates were measured for STAT5, Akt and ERK activity (E). Quantification of pSTAT5 was measured with indicated time periods when lysates were prepared from ruxolitinib wash and DMSO wash (F). Similarly, ruxolitinib washout and DMSO washout samples were incubated for the indicated time period to measure c-Myc, PIM1, PIM2 and ID1 levels (G). SET-2 cells were treated with 1μM ruxolitinib or DMSO for a time period of 45 minutes, ruxolitinib and DMSO were washed out from cells and incubated for a period of 24 hours. Cell proliferation was measured using 3-(4,5-dimethylthiazol-2-yl)-5-(3-carboxymethoxyphenyl)-2-(4-sulfophenyl)-2h-tetrazolium (MTS)- based method. Data is shown as mean ± standard deviation (SD) (n=3). OD – optical density (H). **p<0.01, n.s., not significant, p>0.05 by Student’s t test.

**Figure S12. MPL and ID1 has STAT binding GAS sites:** Promoter region (-1000bp) from the start codon of MPL and ID1 was analyzed for GAS consensus sequence TTCN_(2-4)_GAA. Both genes have one typical GAS sequence (red), other possible non-typical GAS sequence, as mentioned before^17^, were represented in green.

**Figure S13. The type II JAK2 inhibitor (CHZ868) does not induce hyperphosphorylation of the activation loop Tyr1007/1008 and does not show hyperactivation of STAT5 target genes after CHZ868 dissociation:** (A) Ba/F3 cells expressing JAK2-V617F were treated with the indicated concentration of CHZ868 for 3 hours and lysates were prepared and subjected to Western blotting for pJAK2, JAK2, pSTAT5 and STAT5. (B) GSEA analysis identifies Hallmark gene sets enriched in CHZ868-treated compared to DMSO-treated samples. The x-axis represents the -log10(p-value), indicating the statistical significance of enrichment. The y-axis shows the names of the Hallmark gene sets. The color of each bar represents the direction of enrichment, with green indicating upregulation and red indicating downregulation of genes within the corresponding set. (C) Heatmap depicting the log-CPM expression values (Z-score scaled) of selected genes across DMSO-treated and CHZ868-treated experimental conditions. The x-axis represents the experimental group replicates, and the y-axis shows the individual genes. The p-value scale at the left provides a statistical significance reference for interpreting the expression values. (D) Heatmap depicting the log-CPM expression values (Z-score scaled) of selected genes across DMSO-wash and CHZ868-wash experimental conditions. The x-axis represents the experimental groups replicates, and the y-axis shows the individual genes. The p-value scale at the left provides statistical significance reference for interpreting the expression values. (E) Quantification of representative values of PIM2, ID1, MPL and TGM2 between DMSO wash and CHZ868 wash samples. p<0.5 and n.s., not significant.

**Figure S14. Ruxolitinib persistence is circumvented by inhibition of PIM kinases:** Single clones of Ba/F3 cells grown in 96-well plates in the presence of 2μM ruxolitinib alone, 2μM ruxolitinib together with 500nM TP-3654, 4μM ruxolitinib alone, and 4μM ruxolitinib together with TP-3654 were picked and expanded at the indicated concentration. The number of resistant clones per million cells input is shown. Co-treatment of the PIM kinase inhibitor TP-3654 with ruxolitinib prevents the emergence of ruxolitinib resistance.

**Figure S15. Ruxolitinib and Fedratinib persistent HEL cells display upregulation of PIM kinases and ID1:** RNA-seq data from Kong et al. study was extracted from the GEO accession number: GSE190517^18^. Heatmap depicting the log-CPM expression values (Z-score scaled) of selected genes across parental HEL cells and ruxolitinib persistent cells (A); parental HEL cells and fedratinib persistent cells (B). The x-axis represents the experimental groups replicates, and the y-axis shows the individual genes. The p-value scale at the left provides statistical significance reference for interpreting the expression values.

**Figure S16. Ruxolitinib recovery leads to reactivation of JAK-STAT. Chemokine signaling pathways and PIM kinases are indispensable for ruxolitinib-induced hyperproliferation:** The proteomap illustrates the most altered kinases, represented as polygon-shaped tiles, with their mean kinase statistics. Proteins within the same category are color-coded similarly and positioned adjacently to form larger regions. In this case, the activity of all displayed kinases is enhanced in ruxo washout (15min) compared to the ruxolitinib treated group. The size of each polygon is proportional to the magnitude of change (A). Tyrosine and serine/threonine kinases recovered by ruxolitinib recovery in the phylogentic kinome tree. (B) String network (medium confidence (0.400) for kinases influenced by the ruxolitinib recovery (recovered kinases shown in green, blue = reduction of kinase activity, represented by the mean kinase statistic (C). PIM1 and PIM2 were ectopically expressed in Ba/F3 JAK2-V617F cells and treated with 1μM ruxolitinib or DMSO for a time period of 45 minutes. Ruxolitinib and DMSO were washed out from cells and incubated for a period of 24 hours. Cell proliferation was measured using (MTS)- based method. Data is shown as mean ± standard deviation (SD) (n=3). OD – optical density (D). PIM1 and PIM2 were stably knocked down in Ba/F3 JAK2-V617F cells and treated with 1μM ruxolitinib or DMSO for a time period of 45 minutes, Ruxolitinib and DMSO were washed out from cells and incubated for a period of 24 hours. Cell proliferation was measured using (MTS)- based method. Data is shown as mean ± standard deviation (SD) (n=3). OD – optical density (E). ****p<0.0001; **p<0.01, *p<0.01 and n.s., not significant, p>0.05 by Student’s t test.

**Supplementary Information**

**JAK2 hyperphosphorylation is not due to the inhibition of kinase activity of the pseudokinase domain**

Recent data demonstrated that inhibition of the kinase activity of the pseudokinase domain (JH2) leads to an increase in the phosphorylation of the activation loop Tyr1007 and Tyr1008^19^. Thus, we assumed that ruxolitinib treatment might inhibit the kinase activity of the JH2 domain of JAK2. To test this, a phospho-deficient mutant (S523A) and a loss of ATP binding mutant (K581R) were introduced to prevent the kinase activity of the JH2 domain. JAK2 constructs containing V617F, V617F+S523A and V617F+K581R were stably expressed in IL-3–dependent Ba/F3 cells. As described previously, the expression of JAK2-V617F in Ba/F3 cells led to a factor-independent growth^1^. As expected, both JAK2-V617F+S523A and JAK2-V617F+K581R promoted factor-independent growth, and slightly increased cell growth was observed compared to control cells expressing JAK2-V617F (Supplementary Fig. 3A). Consistent with the cell proliferation data, western blot analysis showed enhanced Tyr1007/Tyr1008 phosphorylation in JAK2-V617F+S523A and JAK2-V617F+K581R compared to JAK2-V617F (Supplementary Fig. 3B), which did not alter sensitivity towards ruxolitinib, indicating that mutation of these residues did not alter ruxolitinib binding (Supplementary Fig. 3C, E). Upon ruxolitinib treatment, JAK2-V617F+S523A and JAK2-V617F+K581A expressing cells displayed paradoxical hyperphosphorylation of the JAK2 activation loop and inhibition of STAT5 similar to JAK2-V617F (Supplementary Fig. 3D, F). These results suggest that the JH2 domain kinase activity does not contribute to ruxolitinib-induced paradoxical JAK2 hyperphosphorylation.

**References:**

1. Gorantla SP, Dechow TN, Grundler R, Illert AL, Zum Buschenfelde CM, Kremer M*, et al.* Oncogenic JAK2V617F requires an intact SH2-like domain for constitutive activation and induction of a myeloproliferative disease in mice. *Blood* 2010 Nov 25; **116**(22)**:** 4600-4611.

2. Grundler R, Brault L, Gasser C, Bullock AN, Dechow T, Woetzel S*, et al.* Dissection of PIM serine/threonine kinases in FLT3-ITD-induced leukemogenesis reveals PIM1 as regulator of CXCL12-CXCR4-mediated homing and migration. *J Exp Med* 2009 Aug 31; **206**(9)**:** 1957-1970.

3. Duyster J, Baskaran R, Wang JY. Src homology 2 domain as a specificity determinant in the c-Abl-mediated tyrosine phosphorylation of the RNA polymerase II carboxyl-terminal repeated domain. *Proceedings of the National Academy of Sciences of the United States of America* 1995 Feb 28; **92**(5)**:** 1555-1559.

4. Bray NL, Pimentel H, Melsted P, Pachter L. Near-optimal probabilistic RNA-seq quantification. *Nature Biotechnology* 2016 2016/05/01; **34**(5)**:** 525-527.

5. Durinck S, Spellman PT, Birney E, Huber W. Mapping identifiers for the integration of genomic datasets with the R/Bioconductor package biomaRt. *Nature Protocols* 2009 2009/08/01; **4**(8)**:** 1184-1191.

6. Durinck S, Moreau Y, Kasprzyk A, Davis S, De Moor B, Brazma A*, et al.* BioMart and Bioconductor: a powerful link between biological databases and microarray data analysis. *Bioinformatics* 2005 Aug 15; **21**(16)**:** 3439-3440.

7. Robinson MD, McCarthy DJ, Smyth GK. edgeR: a Bioconductor package for differential expression analysis of digital gene expression data. *Bioinformatics* 2009; **26**(1)**:** 139-140.

8. McCarthy DJ, Chen Y, Smyth GK. Differential expression analysis of multifactor RNA-Seq experiments with respect to biological variation. *Nucleic Acids Res* 2012 May; **40**(10)**:** 4288-4297.

9. Chen Y, Lun AT, Smyth GK. From reads to genes to pathways: differential expression analysis of RNA-Seq experiments using Rsubread and the edgeR quasi-likelihood pipeline. *F1000Res* 2016; **5:** 1438.

10. Chen Y, Chen L, Lun ATL, Baldoni PL, Smyth GK. edgeR 4.0: powerful differential analysis of sequencing data with expanded functionality and improved support for small counts and larger datasets. *bioRxiv* 2024**:** 2024.2001.2021.576131.

11. Ritchie ME, Phipson B, Wu D, Hu Y, Law CW, Shi W*, et al.* limma powers differential expression analyses for RNA-sequencing and microarray studies. *Nucleic Acids Research* 2015; **43**(7)**:** e47-e47.

12. Subramanian A, Tamayo P, Mootha VK, Mukherjee S, Ebert BL, Gillette MA*, et al.* Gene set enrichment analysis: a knowledge-based approach for interpreting genome-wide expression profiles. *Proc Natl Acad Sci U S A* 2005 Oct 25; **102**(43)**:** 15545-15550.

13. Zillikens H, Kasprick A, Osterloh C, Gross N, Radziewitz M, Hass C*, et al.* Topical Application of the PI3Kβ-Selective Small Molecule Inhibitor TGX-221 Is an Effective Treatment Option for Experimental Epidermolysis Bullosa Acquisita. *Front Med (Lausanne)* 2021; **8:** 713312.

14. Liebermeister W, Noor E, Flamholz A, Davidi D, Bernhardt J, Milo R. Visual account of protein investment in cellular functions. *Proc Natl Acad Sci U S A* 2014 Jun 10; **111**(23)**:** 8488-8493.

15. Metz KS, Deoudes EM, Berginski ME, Jimenez-Ruiz I, Aksoy BA, Hammerbacher J*, et al.* Coral: Clear and Customizable Visualization of Human Kinome Data. *Cell Syst* 2018 Sep 26; **7**(3)**:** 347-350.e341.

16. Szklarczyk D, Gable AL, Lyon D, Junge A, Wyder S, Huerta-Cepas J*, et al.* STRING v11: protein-protein association networks with increased coverage, supporting functional discovery in genome-wide experimental datasets. *Nucleic Acids Res* 2019 Jan 8; **47**(D1)**:** D607-d613.

17. Decker T, Kovarik P, Meinke A. GAS elements: a few nucleotides with a major impact on cytokine-induced gene expression. *J Interferon Cytokine Res* 1997 Mar; **17**(3)**:** 121-134.

18. Kong T, Laranjeira ABA, Yang K, Fisher DAC, Yu L, Poittevin De La Frégonnière L*, et al.* DUSP6 mediates resistance to JAK2 inhibition and drives leukemic progression. *Nature Cancer* 2023 2023/01/01; **4**(1)**:** 108-127.

19. Ungureanu D, Wu J, Pekkala T, Niranjan Y, Young C, Jensen ON*, et al.* The pseudokinase domain of JAK2 is a dual-specificity protein kinase that negatively regulates cytokine signaling. *Nat Struct Mol Biol* 2011 Aug 14; **18**(9)**:** 971-976.
