# Supplementary material for: Ruxolitinib mediated paradoxical JAK2 hyperphosphorylation is due to the protection of activation loop tyrosines from phosphatases
Source: Leukemia. 2025 Apr 23;39(7):1678–91. doi: 10.1038/s41375-025-02594-7 (PMC12208895; doi:10.1038/s41375-025-02594-7)
Supplement: Supplementary file 2 — PamGene _DMSO vs Ruxo_PTK [file 41375_2025_2594_MOESM2_ESM.pdf]

Kinase Name	Mean Specificity Score	Mean Kinase Statistic	SD Kinase Statitistic
Syk	2.33600311912668	-4.18321153376706	0.0754743402604857
Lck	1.98655883795587	-4.65247294264238	0.098655183422995
Lyn	1.68456574424342	-4.6548093826802	0.0985339836134093
BLK	1.58770444977358	-4.63295667872457	0.23604038204675
Src	1.54651027875822	-4.25386846221497	0.182730081025404
Fgr	1.32234943125512	-4.91129902245162	0.413053800884761
Yes	1.3012387074316	-4.48721537035498	0.25497877652482
FRK	1.24933598045747	-4.14574319663872	0.0794876859578212
RYK	1.05041298346043	-4.91827820020053	0.419909144576133
Srm	1.00480390732726	-4.12878553035056	0.0375813719902373
HCK	0.926807437148502	-4.0960888195964	0.25727102051635
HER3	0.860070045632407	-4.01007545787665	0.231095127332502
Fyn	0.767189025000153	-4.26986042192541	0.218409173994914
ZAP70	0.715577934217703	-3.93123399590545	0.0965683320639907
ALK	0.697895199163449	-3.98347425356515	0.0973535987487788
Arg	0.672543318389662	-3.97848787349218	0.2095229122893
Ron	0.64348532495724	-4.31385135720142	0.29827231564025
Mer	0.623962401578508	-3.92415535438927	0.109445121214804
Abl	0.587133356535306	-3.92423107185278	0.203870095395421
InSR	0.536217312159903	-3.97143561582843	0.0681627392739082
Axl	0.535628382310314	-3.89481352472316	0.112800971427901
TEC	0.464516153649328	-3.86287982292931	0.115409788900976
EphA4	0.427621376248391	-4.07291875480339	0.537348405537887
LTK	0.413812976339425	-3.89391396385387	0.102332240394781
IGF1R	0.395935929779066	-3.84846751160988	0.208071321401343
Fes	0.361669809261396	-3.84633300244458	0.353411831907852
BTK	0.343994202066116	-3.78756114076239	0.190242515691194
TRKA	0.302333500298029	-3.68065554448967	0.331256170491354
PDGFR[alpha]	0.287404020279342	-3.66604628722805	0.25152633691594
FLT3	0.271642193078549	-3.44789895742162	0.593051877326794
TRKC	0.259459260068188	-3.70167340021927	0.0768205855640735
EphA1	0.254982361694419	-3.55435952294576	0.0718326725071693
CCK4/PTK7	0.252023646007345	-3.57297187552228	0.0316290236708281
FmS/CSFR	0.249191466320526	-3.58389018913318	0.336593226037227
CTK	0.238519423618428	-3.68238249411779	0.102812380939058
FGFR2	0.238225115456609	-3.61481202867673	0.157735442113361
DDR1	0.233544321802673	-3.32926540157938	0.664175999894646
TRKB	0.223299819622951	-3.65052108370659	0.102116477154984
TXK	0.213343819602235	-3.59168283423612	0.206110077289426
Ret	0.2027038028801	-3.54350899326879	0.223814775243787
Etk/BMX	0.189923149659157	-3.53282149048336	0.298886276733599
CSK	0.186191506479276	-3.64818029556869	0.106131281472476
FAK1	0.181812110459229	-3.60471932251352	0.175158702057318
EphA8	0.177312443618707	-3.19220526216498	0.211418681707545
FAK2	0.17276192283326	-3.58991570683455	0.144413554741159
Met	0.16270809300997	-3.60273476725742	0.0910859520537411
JAK1~b	0.153633090099557	-3.19068970306245	0.225875291040753
KDR	0.145487185082816	-3.49709286471463	0.0950195374291991
EphA2	0.141979139117007	-3.20884801186433	0.27414604681966

ROR1	0.136677139879544	-2.87267684247893	0
HER2	0.134345641362741	-3.51240733819332	0.163647034054347
Kit	0.133479106910888	-3.35972196907697	0.361712717565122
EphA5	0.130929128839783	-3.08883245286881	0.0541211833230875
IRR	0.130113113984226	-3.09525526305672	0.126446821076783
Fer	0.115897867284114	-3.27415836004363	0.279782403533013
Tyro3/Sky	0.114013523178699	-3.5530829086102	0.106931781344062
EphA7	0.107280947310995	-2.74630926002702	0.138199840797333
Lmr1	0.106819646633441	-2.66056824684693	0
PDGFR[beta]	0.0952902593905317	-3.24348228956995	0.13933949915621
Brk	0.09141011377647	-3.56549543438684	0.101464025271602
EphA3	0.0894509370115552	-2.75807260971068	0.220298696360327
EphB3	0.0893755951107988	-2.52904058435057	NA
FLT4	0.0841090584868242	-3.14254485561831	0.27128598440309
EphB4	0.0793803484016174	-2.55788771394531	0
HER4	0.0746686997519651	-3.30816157798386	0.242523998153637
EphB1	0.0622620633539401	-2.47474786096077	0.314017226329542
FGFR1	0.0618721109188761	-3.22721240500522	0.0791964799690521
FGFR3	0.0559779907138308	-3.04973278584865	0.215924391536146
JAK2	0.0355805561707858	-3.21324898963213	0.0847423161659121
ITK	0.0271637032624587	-3.24908632540386	0.0900990900907625
EGFR	0.0189740751669089	-3.30709988016666	0.170486441462392
EphB2	0.0145756159154622	-1.9501756745667	0
FGFR4	0.012232256783071	-2.93456772411517	0.259592770878288
Tyk2	0.00427312560145905	-1.87188405062641	0
JAK3	0.00290577321117519	-2.01565167239377	0.0033636781366789
FLT1	0.00252686788962219	-2.68419558164545	0.18331615881461

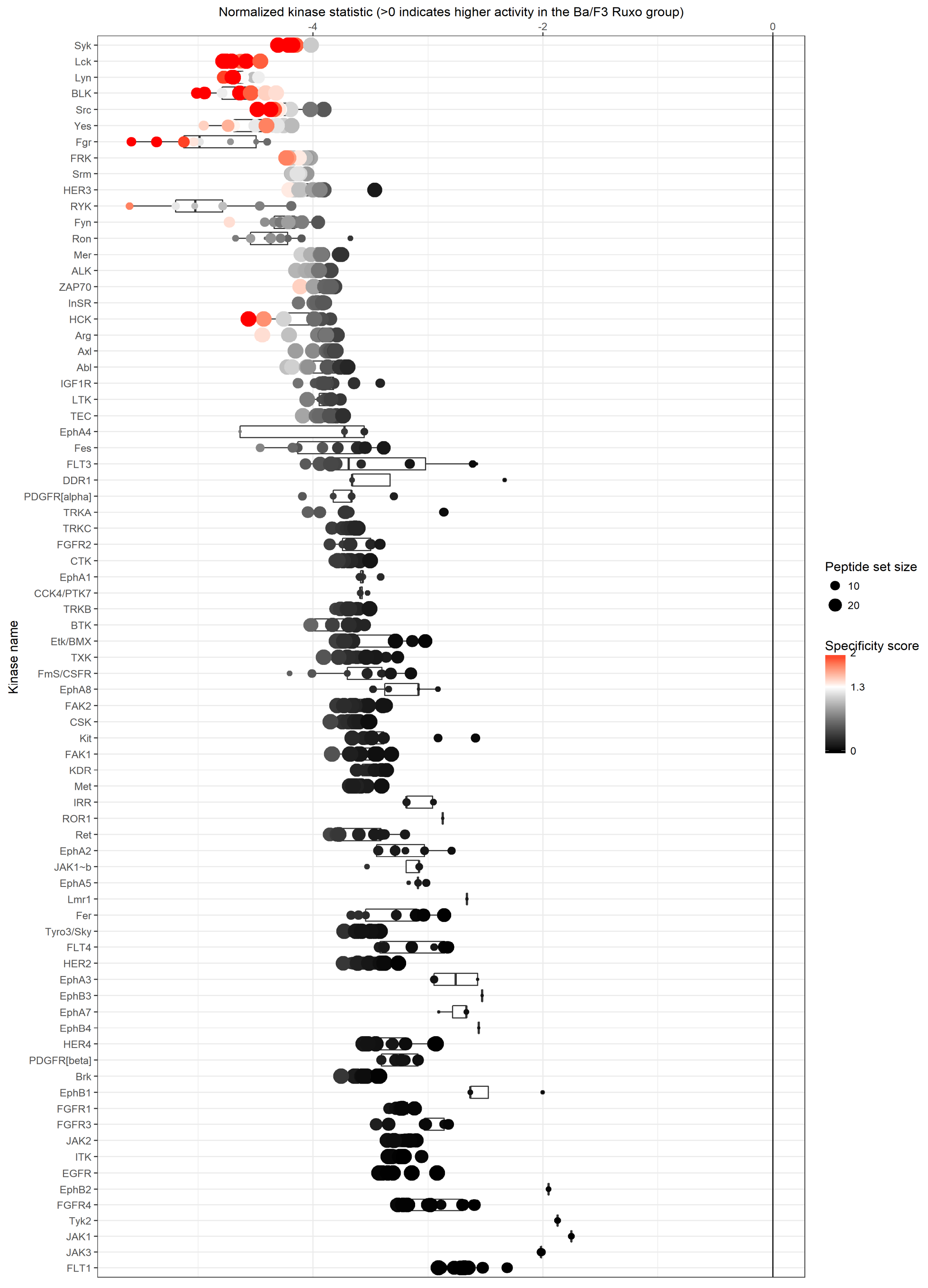

Normalized kinase statistic (>0 indicates higher activity in the Ba/F3 Ruxo group)
-4
-2
0
Kinase name
Syk
Lck
Lyn
BLK
Src
Yes
Fgr
FRK
Srm
HER3
RYK
Fyn
Ron
Mer
ALK
ZAP70
InSR
HCK
Arg
Axl
Abl
IGF1R
LTK
TEC
EphA4
Fes
FLT3
DDR1
PDGFR[alpha]
TRKA
TRKC
FGFR2
CTK
EphA1
CCK4/PTK7
TRKB
BTK
Etk/BMX
TXK
FmS/CSFR
EphA8
FAK2
CSK
Kit
FAK1
KDR
Met
IRR
ROR1
Ret
EphA2
JAK1~b
EphA5
Lmr1
Fer
Tyro3/Sky
FLT4
HER2
EphA3
EphB3
EphA7
EphB4
HER4
PDGFR[beta]
Brk
EphB1
FGFR1
FGFR3
JAK2
ITK
EGFR
EphB2
FGFR4
Tyk2
JAK1
JAK3
FLT1
Peptide set size
10
20
Specificity score
2
1.3
0
