# Supplementary material for: Ruxolitinib mediated paradoxical JAK2 hyperphosphorylation is due to the protection of activation loop tyrosines from phosphatases
Source: Leukemia. 2025 Apr 23;39(7):1678–91. doi: 10.1038/s41375-025-02594-7 (PMC12208895; doi:10.1038/s41375-025-02594-7)
Supplement: Supplementary file 3 — PamGene_DMSO vs Ruxo_STK [file 41375_2025_2594_MOESM3_ESM.pdf]

Kinase Name	Mean Specificity Score	Mean Kinase Statistic	SD Kinase Statitistic	
Pim2	1.62187234595847	-1.05034211437251	0.0506204408138527	
PKG1	1.60259748670778	-1.03240836921351	0.0149097640841584	
PKC[zeta]	1.54131800905161	-1.34559544241613	0.0353333175713595	
PKC[theta]	1.49756422584041	-1.13201840826194	0.0309020632925669	
AurB/Aur1	1.22024296221227	-1.52892316903098	0.24517011135366	
CHK1	1.02608429893778	-1.20174235356415	0.0641199763306391	
PKC[gamma]	1.01265079227448	-1.1132240332696	0.075301166852925	
DAPK2	1.01134264556119	-1.42721408268517	0.319293609877712	
PKC[epsilon]	0.983641754490723	-1.04040477416457	0.0611345524837265	
PKC[eta]	0.973825299219304	-1.11099435453596	0.0693140397098163	
PAK1	0.973677294357871	-1.27337918276113		0
PKC[beta]	0.946161569113698	-1.16269686137364		0
PKC[alpha]	0.923142935874096	-0.987305560947894	0.0235198927322008	
PKN1/PRK1	0.898597526487261	-1.24004516119348	0.0226584286637247	
NuaK1	0.895921018562621	-1.34915071205226	0.0603996068161999	
PKC[delta]	0.759585488548799	-0.986725246742701	0.0118130185194804	
DAPK3	0.72588325957368	-1.24672464173521	0.0627582141281505	
RSKL1	0.664760908079358	-1.18328765975258	0.0383705159711489	
MAPKAPK2	0.626996352835512	-0.930620683439575	0.0341242064648599	
PKA[alpha]	0.597947201683475	-0.879577758679417	0.0584230516438456	
PKC[iota]	0.55251414492892	-0.959654904684249	0.0677910997997757	
Pim1	0.541479521724946	-0.876088394949985	0.0144277999917636	
MSK2	0.532849657805894	-1.06195548675736		0
PRKX	0.493838750300337	-0.901449589115421	0.0471911492154985	
CDK10	0.484435375618736	-0.973215282717393	0.232404866117877	
CaMK2[alpha]	0.478539935279686	-1.01340116327574		0
AlphaK1	0.472372011166251	-0.972870103430295		0
p70S6K	0.46750276475445	-0.917333879419997	0.053475613755569	
ICK	0.439842835838578	-0.983753296084104		0
COT	0.435722915157827	-0.961101485637061		0
RSK1/p90RSK	0.422994471150259	-0.944234141561634		0
Pim3	0.413500300776197	-0.860683122087931	0.0283556898925803	
ERK1	0.407325424962013	-0.890765937488237	0.0839292073789961	
RSK2	0.406821882260239	-0.912769245995422		0
MAPKAPK3	0.395457836782929	-0.871096599767037	0.0340603288859797	
DCAMKL1	0.388627991956026	-0.860394788555849	0.285034861249255	
PFTAIRE1	0.385355330828393	-0.919576858598499		0
MSK1	0.373771793043878	-0.894263374547457		0
BRAF	0.371323328961654	-0.904207818815314	0.0191941702390336	
ARAF	0.356154489593452	-0.894593055124595		0
ERK7	0.348721986001856	-0.851997848514551	NA	
AurA/Aur2	0.343087661591748	-0.861676238906154	0.00567179680212315	
PFTAIRE2	0.339060895923554	-0.871184506598421	0.0333073520848645	
ADCK3	0.33114057703014	-0.856572647992238	0.135889708902185	
CK2[alpha]1	0.299974032839105	-0.812232777370315	0.0450803993261324	
PKD1	0.290302997329136	-0.827626816214473		0
SGK2	0.256919944823559	-0.814127910284382	0.0585217411076112	
MAPK14	0.255375190824745	-0.805363518724102	0.128716959181548	
p38[delta]	0.253927174416074	-0.803961522369184	0.101787051652237	

IKK[alpha]	0.247205378442074	-0.752517504511673	0.00895111524186881	
PKG2	0.226592408485471	-0.80115887167414	0.0610903834600109	
p70S6K[beta]	0.215602441007916	-0.767560893474769	0.068809294264964	
GSK3[alpha]	0.213996529759425	-0.708298774110759	0.0569008714689849	
JNK2	0.208232968463381	-0.789536320669117	0.0337918773320813	
ROCK1	0.200610795504412	-0.573523332261915	0.419628541367321	
CK1[alpha]	0.198278178318496	-0.717338644423807	0.160216311891937	
CDK5	0.19606643158807	-0.737197250782297		0
CK1[epsilon]	0.18309616062434	-0.653268938361053	NA	
IKK[epsilon]	0.182034483276607	-0.70078524762567	0.0744532496392434	
GSK3[beta]	0.181789387420752	-0.680041355577677	0.0360040463166109	
HGK/ZC1	0.177634291450945	-0.651592541313417	0.143060644674281	
RSK3	0.167551923457304	-0.717987144150795		0
p38[beta]	0.161320753949267	-0.640744373855771	0.101241322617808	
CaMK4	0.158410117381745	-0.719257498061795	0.0560261184334155	
RAF1	0.157517505163459	-0.676210435380168	0.112047618196455	
CHK2	0.157377152501257	-0.727032956299381	0.0823729018473861	
ATR	0.156836290132718	-0.676876373839897	0.0629506341337935	
TBK1	0.144927449107976	-0.586609084366249	0.0743913466184132	
ERK2	0.140252509503855	-0.714524108806464	0.0974263615647622	
CDK3	0.136308086026242	-0.633165763023117	0.167138376679447	
JNK3	0.13355264314653	-0.751847625420401	0.00932900916958958	
p38[gamma]	0.133087690866621	-0.653567822899497	0.0598971957885604	
CDC2/CDK1	0.126769006922929	-0.706905950598727	0.0481340446177173	
JNK1	0.126161088391683	-0.749464224809208	0.0061043841925818	
PRKY	0.108998725187666	-0.59421770555788	0.148829286314385	
CDK2	0.104812759422364	-0.691964617226534	0.0260024520574949	
SGK1	0.0968137340291093	-0.475116242357846		0
Akt1/PKB[alpha]	0.0962111938168278	-0.692759669585601	0.0936720351788534	
CDKL1	0.0841833902356777	-0.483758227821457	0.145495943605201	
ERK5	0.0784778395226331	-0.571721025310686	0.101955314310535	
CDKL5	0.0733985645873525	-0.489088102545058	0.074796506803082	
PCTAIRE2	0.0720689599917761	-0.371845719130728	0.161894825076887	
SgK307	0.0599402407925518	-0.387671028864711	0.134148018858478	
ROCK2	0.0532450760297517	-0.37693364550357	0.141286340674676	
ANP[alpha]	0.0511618242720149	-0.653058324150757	0.0456204445948322	
RSKL2	0.0501556307570546	-0.344135998812628	0.0116551137997644	
Akt2/PKB[beta]	0.049120860707137	-0.644199426415737	0.0737771776395484	
CDK9	0.0335626608769781	-0.386382386852801	0.122775135697524	
mTOR/FRAP	0.0265763799251984	-0.260235519343692	0.102479944773021	
CDK4	0.0226281552889033	-0.299723160830219	0.0275511169751381	
AMPK[alpha]1	0.0217178797068951	-0.420859120655931	0.0204179414097171	
IKK[beta]	0.0207867630750422	-0.153119467628414	0.171964395692063	
CDKL2	0.0127929971115133	-0.104595365217908	0.0606333994079058	
CDK7	0.00987542778783747	-0.389271052231612	0.0397841210687278	
CDK11	0.00966316159966018	-0.0857042198589977		0
CDK6	0.00877836074656036	-0.117383488327434	0.0118797256756637	
TNIK/ZC2	0.00393010441253716	0.0351993466050704		0

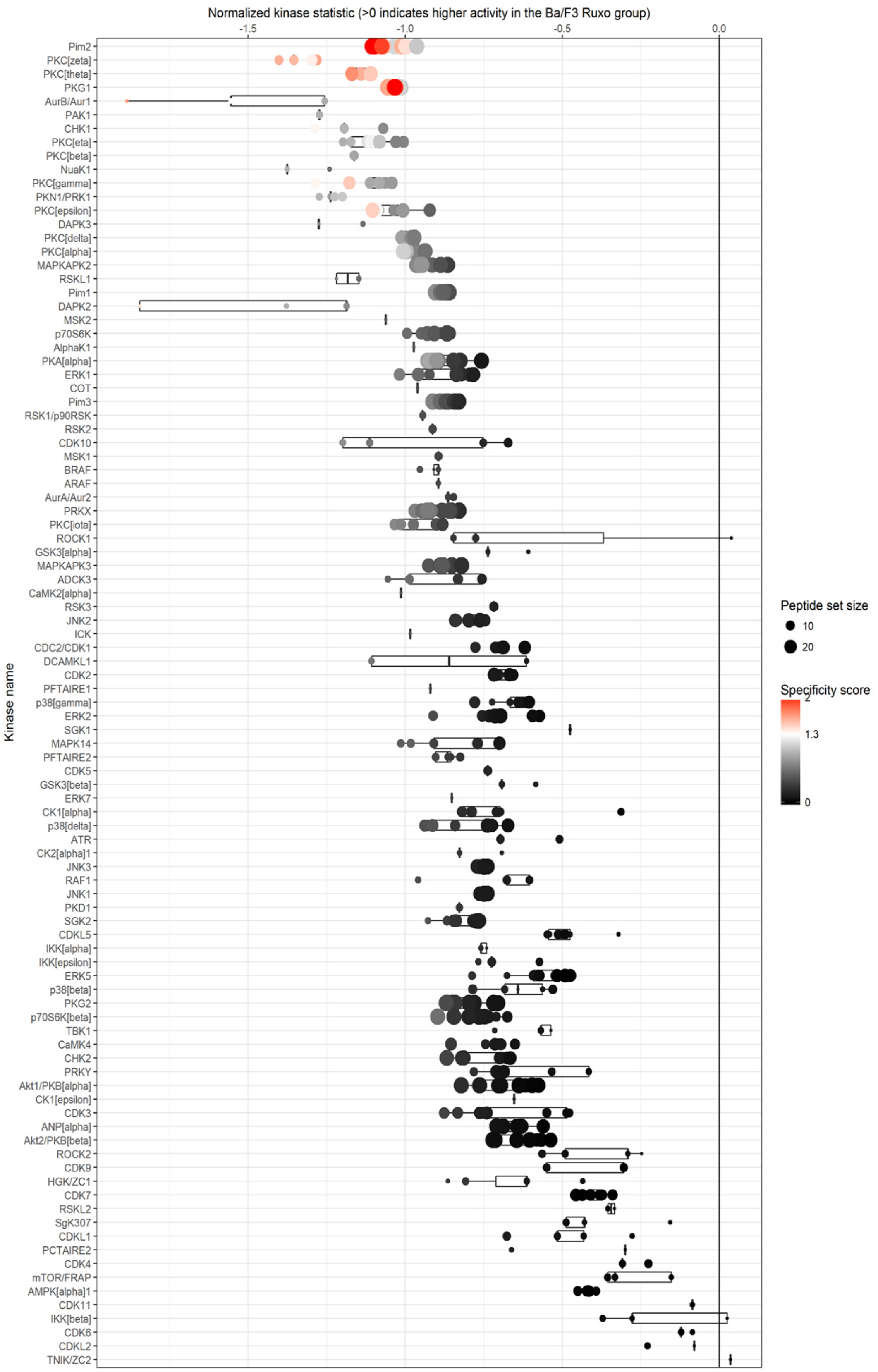

Normalized kinase statistic (>0 indicates higher activity in the Ba/F3 Ruxo group)
-1.5
-1.0
-0.5
0.0
Kinase name
Pim2
PKC[zeta]
PKC[theta]
PKG1
AurB/Aur1
PAK1
CHK1
PKC[eta]
PKC[beta]
NuaK1
PKC[gamma]
PKN1/PRK1
PKC[epsilon]
DAPK3
PKC[delta]
PKC[alpha]
MAPKAPK2
RSKL1
Pim1
DAPK2
MSK2
p70S6K
AlphaK1
PKA[alpha]
ERK1
COT
Pim3
RSK1/p90RSK
RSK2
CDK10
MSK1
BRAF
ARAF
AurA/Aur2
PRKX
PKC[iota]
ROCK1
GSK3[alpha]
MAPKAPK3
ADCK3
CaMK2[alpha]
RSK3
JNK2
ICK
CDC2/CDK1
DCAMKL1
CDK2
PFTAIRE1
p38[gamma]
ERK2
SGK1
MAPK14
PFTAIRE2
CDK5
GSK3[beta]
ERK7
CK1[alpha]
p38[delta]
ATR
CK2[alpha]1
JNK3
RAF1
JNK1
PKD1
SGK2
CDKL5
IKK[alpha]
IKK[epsilon]
ERK5
p38[beta]
PKG2
p70S6K[beta]
TBK1
CaMK4
CHK2
PRKY
Akt1/PKB[alpha]
CK1[epsilon]
CDK3
ANP[alpha]
Akt2/PKB[beta]
ROCK2
CDK9
HGK/ZC1
CDK7
RSKL2
SgK307
CDKL1
PCTAIRE2
CDK4
mTOR/FRAP
AMPK[alpha]1
CDK11
IKK[beta]
CDK6
CDKL2
TNIK/ZC2
Peptide set size
10
20
Specificity score
2
1.3
0
