# Supplementary material for: Ruxolitinib mediated paradoxical JAK2 hyperphosphorylation is due to the protection of activation loop tyrosines from phosphatases
Source: Leukemia. 2025 Apr 23;39(7):1678–91. doi: 10.1038/s41375-025-02594-7 (PMC12208895; doi:10.1038/s41375-025-02594-7)
Supplement: Supplementary file 4 — PamGene_Ruxo vs Ruxo wash_PTK [file 41375_2025_2594_MOESM4_ESM.pdf]

Kinase Name	Mean Specificity Score	Mean Kinase Statistic	SD Kinase Statitistic
Syk	2.63207444974402	0.711834417829092	0.0270415788169997
Src	2.33415875670229	0.857904717083779	0.03099322585907
Lck	2.29814166842328	0.943024116635832	0.042339279748938
HER3	1.78290240494477	0.756264629862209	0.0715749566768885
JAK3	1.77166067830546	1.32525644199642	0.156190983576714
Lyn	1.64971639116563	0.934284238946181	0.0519363614803654
ZAP70	1.5458344629163	0.651419013295723	0.0343255154534179
BLK	1.43701611563462	0.881104308445367	0.0680584219650667
Fgr	1.38358797316834	1.14701948802103	0.189530860443654
HCK	0.990367050301453	0.704331052609519	0.0753388980650902
FGFR3	0.874899819036464	0.822466716553045	0.15613903336999
FLT4	0.865320433878005	-0.761603542766681	0.320683593936393
Ron	0.824094725179944	0.940526371900589	0.149310573695273
Etk/BMX	0.817446891028434	0.626831092701394	0.0906949402970193
FRK	0.793157043009617	0.575868197557578	0.0395898149994387
ALK	0.750706454515512	0.554313253463442	0.0913435549161368
FGFR2	0.736607335058784	0.76793069653608	0.112015348175826
InSR	0.682229478940919	0.63003097611113	0.0393692601994836
FGFR1	0.668672255321978	0.66903881331145	0.200206950427915
TRKC	0.65337308547323	0.627557520730414	0.0874886365159629
Fyn	0.555642473838648	0.618050536552395	0.0722453222488599
Yes	0.555535428602632	0.591386039144098	0.0698876843008844
Tyk2	0.523248061873146	0.820583473831014	0
Axl	0.514967328997159	0.497972430144085	0.0430429515731338
EphA3	0.51470262088776	0.821481540083661	0.0328868380722494
LTK	0.507953909296642	0.606233504987068	0.184187835040793
BTK	0.50781614739433	0.567432707936045	0.109409357209845
EphA8	0.506142230837176	0.839954709758521	0.109254320257143
IGF1R	0.497146522606742	0.552188006358473	0.114111546678312
Met	0.487062174959273	0.527066915141142	0.0307734970739813
IRR	0.485920688875926	0.68185117871454	0.251145900145346
TEC	0.452585241041957	0.486621146295194	0.0897730320141294
CTK	0.449168599811542	0.525570468500227	0.171390789964328
EphA5	0.435154936552293	0.69411454789964	0.0777318821611028
FAK1	0.422146286580995	0.47486534142208	0.0499977420841968
EphA7	0.420227378826235	0.77548413354928	0.0898963965396866
FLT1	0.401761272605942	-0.509501521715748	0.163130513765685
TRKA	0.376187062747512	0.510257137590675	0.0774962620592124
Brk	0.357991781550766	0.455038162631822	0.0509131921171863
EphA2	0.344933043390225	0.571501554081356	0.158196101697435
Arg	0.343421600368361	0.443603696392526	0.0658619525463413
Abl	0.319709969377347	0.453178663809602	0.0682579986599569
Kit	0.305429417225636	0.484883178330822	0.137458902579874
TRKB	0.304923101146434	0.447621932312844	0.0620375567162462
TXK	0.300061046865296	0.393747312724163	0.12744956676528
FGFR4	0.272045460370519	0.431805470411398	0.10753617736054
JAK2	0.262597083244745	0.410096482150379	0.0824834171649716
FAK2	0.254036086498217	0.411896227504043	0.0556874366311218
Fer	0.222739207428083	-0.348330314869742	0.16905235670578

JAK1~b	0.213743036308399	-0.423251925507344	0.0189629605455489	
ROR1	0.190453334686629	0.47463350701957		0
EphB2	0.185128071600244	0.445024951902561		0
FLT3	0.172363870459707	-0.245304737223182	0.333960225648221	
RYK	0.17228112027573	0.32281835891698	0.132415540449547	
Mer	0.168617230716603	0.38026472953598	0.0460389193743368	
EphA1	0.151551944098208	0.33034187910943	0.121648145415479	
Ret	0.136247265930936	0.081594834224213	0.28967013500997	
PDGFR[alpha]	0.131140593986832	0.0948156533548408	0.294942475739682	
EphB4	0.122062572163754	-0.335910812488109		0
Tyro3/Sky	0.115032202573055	0.328704018645682	0.0425908491953858	
EphA4	0.101496006425149	0.232088306806561	0.175352326263788	
Fes	0.088259879861453	0.0339631166958124	0.251989634283723	
DDR1	0.0849576396232251	-0.219628523283193	0.233747241551608	
FmS/CSFR	0.0758822403615424	-0.131416429233499	0.173662198481602	
Lmr1	0.0758706171013435	0.209605773063367		0
HER4	0.0737676416569919	0.161898912360334	0.205422792059304	
Srm	0.0626841302133886	0.259417034226702	0.0398610412944456	
EGFR	0.0543448360979043	0.252682734294065	0.0834064941985694	
EphB3	0.0506099933550872	0.14432780671353	NA	
CCK4/PTK7	0.0465072604258124	-0.0302853232476336	0.174385246321775	
PDGFR[beta]	0.0439523416109647	0.102661582300182	0.0891429420141336	
KDR	0.0420754515746877	-0.113070903738693	0.0679979166571959	
HER2	0.0347182342538374	0.137933964418365	0.0514064035710557	
EphB1	0.0272526692130082	-0.00885893435578457	0.114751490509157	
CSK	0.0174128943402817	0.15959930871962	0.0429861764133701	
ITK	0.014896618438945	0.0572298961520131	0.111696337990421	

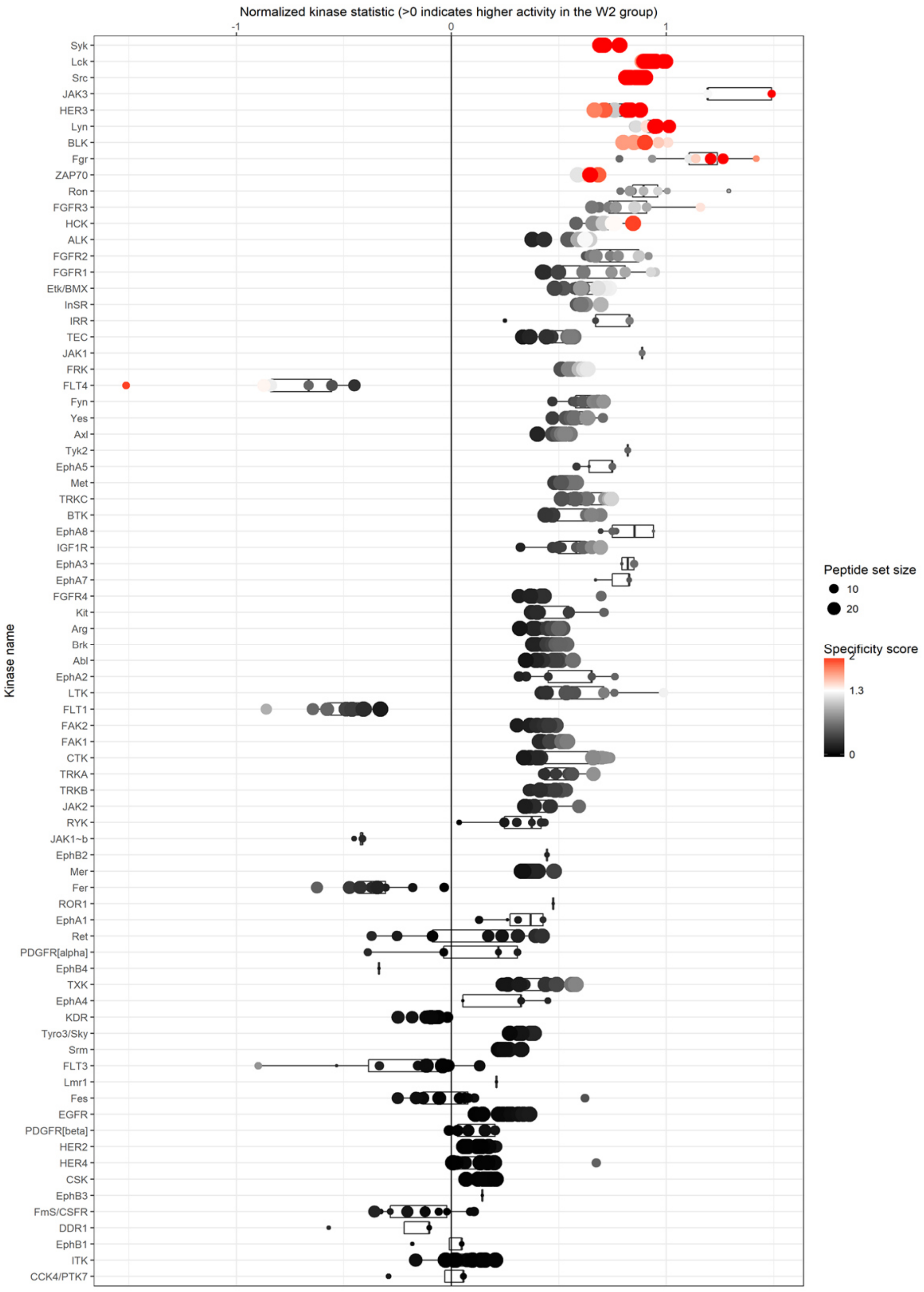

Normalized kinase statistic (>0 indicates higher activity in the W2 group)
-1
0
1
Kinase name
Syk
Lck
Src
JAK3
HER3
Lyn
BLK
Fgr
ZAP70
Ron
FGFR3
HCK
ALK
FGFR2
FGFR1
Etk/BMX
InSR
IRR
TEC
JAK1
FRK
FLT4
Fyn
Yes
Axl
Tyk2
EphA5
Met
TRKC
BTK
EphA8
IGF1R
EphA3
EphA7
FGFR4
Kit
Arg
Brk
Abl
EphA2
LTK
FLT1
FAK2
FAK1
CTK
TRKA
TRKB
JAK2
RYK
JAK1~b
EphB2
Mer
Fer
ROR1
EphA1
Ret
PDGFR[alpha]
EphB4
TXK
EphA4
KDR
Tyro3/Sky
Srm
FLT3
Lmr1
Fes
EGFR
PDGFR[beta]
HER2
HER4
CSK
EphB3
FmS/CSFR
DDR1
EphB1
ITK
CCK4/PTK7
Peptide set size
10
20
Specificity score
2
1.3
0
