# Supplementary material for: Ruxolitinib mediated paradoxical JAK2 hyperphosphorylation is due to the protection of activation loop tyrosines from phosphatases
Source: Leukemia. 2025 Apr 23;39(7):1678–91. doi: 10.1038/s41375-025-02594-7 (PMC12208895; doi:10.1038/s41375-025-02594-7)
Supplement: Supplementary file 5 — PamGene_Ruxo vs Ruxo wash_STK [file 41375_2025_2594_MOESM5_ESM.pdf]

Kinase Name	Mean Specificity Score	Mean Kinase Statistic	SD Kinase Statitistic
PKG1	2.69897000433602	0.236414375244297	0.0433067243589723
PKG2	2.14326027950417	0.207245735839498	0.00954757323102063
CDKL2	1.36229158660367	-0.631557630153492	0.06220959242645
CK2[alpha]1	1.22362515623285	0.519472878742943	0.0240441625625311
CK1[epsilon]	1.15490195998574	0.620373125737966	NA
CaMK4	1.05381999336315	0.238858756631808	0.0244319535529982
PKA[alpha]	0.998754824270866	0.120503610778016	0.00869135987565675
IKK[epsilon]	0.992800071458328	0.338136952642073	0.0844914446043831
IKK[alpha]	0.975377630172391	0.434360993968676	0.0159242369902184
p70S6K[beta]	0.873299066015577	0.160237790159964	0.0173229892886795
RSK1/p90RSK	0.807688455031698	0.285774895519936	0
CDK9	0.801318217936214	-0.264884301500204	0.0326988516607758
p38[beta]	0.793327034717239	0.284037383457164	0.160933997349924
PFTAIRE1	0.732916139434945	0.346226712720769	0
ROCK1	0.724807326182181	-0.309444893595948	0.0326431143096977
PRKX	0.713006322777313	0.127485486693997	0.0226070793247841
ARAF	0.708118880990244	0.310240308623653	0
PKD1	0.691296553857761	0.255627229553036	0
Pim2	0.688060067680528	0.112280821706599	0.0136633128807799
ERK5	0.67452654721245	-0.165505606478816	0.0763496028685696
BRAF	0.631262657880917	0.283228556652515	0.0586592179724248
RSKL2	0.629877499394981	-0.296340327538696	0.038442952191023
SGK1	0.609880765045061	-0.300029692526682	0
CDK11	0.60895321554183	-0.277570622070174	0
AurA/Aur2	0.582005243331661	0.24015837851889	0.00427164629560449
ADCK3	0.576132009507758	-0.190318058220377	0.0270541197269127
ROCK2	0.554757202498718	-0.247409146992805	0.0589893877770885
RSK3	0.551760470814627	0.169992544052295	0
ANP[alpha]	0.540542418660681	0.118189544963594	0.00754782142139768
PFTAIRE2	0.538828088851113	0.204612867431748	0.0246630009589651
CHK2	0.516764021965024	0.120718497310664	0.0170960266961855
TBK1	0.505435441095314	0.234533229446583	0.0268093350908755
PKC[alpha]	0.48941749723352	0.0994514139536239	0.0155816364562449
Pim1	0.472434019132382	0.0720218159113606	0.0226089768791718
CaMK2[alpha]	0.469896235497886	0.240024381268468	0
p38[delta]	0.45948273898283	0.105466690367302	0.119420405601223
Pim3	0.454017034822969	0.0730765554179959	0.0151503803926615
PKC[beta]	0.434326536419592	-0.146347581683618	0
MAPK14	0.425420413141092	0.130292685003784	0.082342461099526
PAK1	0.41413638866964	0.171282756021594	0
Akt1/PKB[alpha]	0.398648231458116	0.0890432501703526	0.0222797666563246
Akt2/PKB[beta]	0.317962425580509	0.0745216408502796	0.0157622744682173
p38[gamma]	0.316662720478742	0.018792941840905	0.128851967485808
ERK1	0.287266039149736	0.0553312400877113	0.0919986544539769
MSK1	0.278580031274163	0.109661231010173	0
PKN1/PRK1	0.278102336300898	-0.125682736190338	0.0173626651453767
PKC[delta]	0.275786127993499	0.0702349707749199	0.0338669325243542
ICK	0.269797053031687	0.151764208360762	0
NuaK1	0.263866495504552	0.141664948831293	0.0178375666840892

ERK2	0.261388289948631	0.0106949501667725	0.110863356366016	
PRKY	0.261321848685103	-0.00432894419020032	0.10095913182862	
CDK10	0.26089986928246	0.119910208459611	0.0586698651241364	
PCTAIRE2	0.258820145084971	0.142051902999049	0.0501345348570996	
AlphaK1	0.258346169455964	0.144655876268192		0
RAF1	0.233927150387487	0.100965064727848	0.0209123136964952	
CDKL1	0.228810542751258	0.10510897012795	0.0871873332168354	
CHK1	0.203398229148992	-0.0783589763977223	0.0440034458648577	
DAPK3	0.200592682111246	0.110712788900598	0.0111980069989835	
RSKL1	0.193728304284916	-0.00469057244577774	0.116739198692218	
CK1[alpha]	0.185527475609302	0.0559949749221163	0.0596689347226779	
mTOR/FRAP	0.173333305268552	0.090945998574629	0.0331900352191975	
CDK2	0.164446740204421	-0.0538199449870224	0.022468958039878	
HGK/ZC1	0.163533013608731	0.0857250914192016	0.0351735922301998	
AMPK[alpha]1	0.159166002401544	0.0540755056272341	0.0481905106599149	
PKC[eta]	0.15761244507598	-0.0379777545601668	0.0700982836622533	
PKC[zeta]	0.156874316472063	-0.0624575429325042	0.0308741762398773	
p70S6K	0.154336785827725	0.0281261289254437	0.0440176297703181	
PKC[epsilon]	0.152345235407662	0.0450047947493119	0.0325952157150942	
SGK2	0.137359977817878	-0.0052143692308746	0.0562125784810708	
CDK6	0.125150959682684	-0.0622701422738089	0.0807376799236368	
AurB/Aur1	0.123481496517276	-0.0668016656346574	0.0587315259166551	
IKK[beta]	0.123328614753641	0.0614999140425869	0.0511070414073845	
RSK2	0.120921887715681	0.0544234564862953		0
CDK7	0.118008158626654	-0.0300541138996371	0.0568442381829592	
CDK4	0.112650365599118	-0.056414964775757	0.00992538095869716	
MAPKAPK3	0.105754442254034	0.0276752966299573	0.0169918572410954	
GSK3[alpha]	0.105285875322898	-0.0519987766537726	0.10545439301709	
CDK3	0.102963265066706	0.00811599252541404	0.0473741703984349	
COT	0.0989352800398506	-0.0626544098568366		0
DCAMKL1	0.094183244212961	-0.0533744183347295	0.0202383966726995	
MAPKAPK2	0.09213167338783	0.0271842101034503	0.0102399602096552	
GSK3[beta]	0.0886008770123839	0.0167636633276475	0.0569047972130288	
ATR	0.0852439409985405	-0.0403274955403421	0.0183143046325075	
JNK1	0.0846969643606219	-0.0257768068894852	0.00720186329293578	
JNK3	0.0799371790024282	-0.0208099723533843	0.0154254080116763	
CDKL5	0.0777982188185259	-0.00589724225577941	0.0512919825187787	
MSK2	0.0765686183155234	-0.0509016576629057		0
CDC2/CDK1	0.0665942131971316	0.0115820390406441	0.0275868981283523	
SgK307	0.0653297770563436	-0.036243249215356	0.0304415267379647	
PKC[theta]	0.0607852655043241	0.0180451733499325	0.0177025381100951	
DAPK2	0.0538285756169885	0.0332604218992873	0.0192282325499944	
PKC[gamma]	0.0529801056925358	-0.0118841291074428	0.0491089817558197	
PKC[iota]	0.0526315874978559	0.0177211040780305	0.014755738581284	
TNIK/ZC2	0.0474907692905204	0.0306637580896781		0
JNK2	0.0396065255305376	-0.00610103411523116	0.0243682548153852	
CDK5	0.0186453917850061	-0.00944595040220368		0
ERK7	0.0186344909214556	0.0103340876413808	NA	

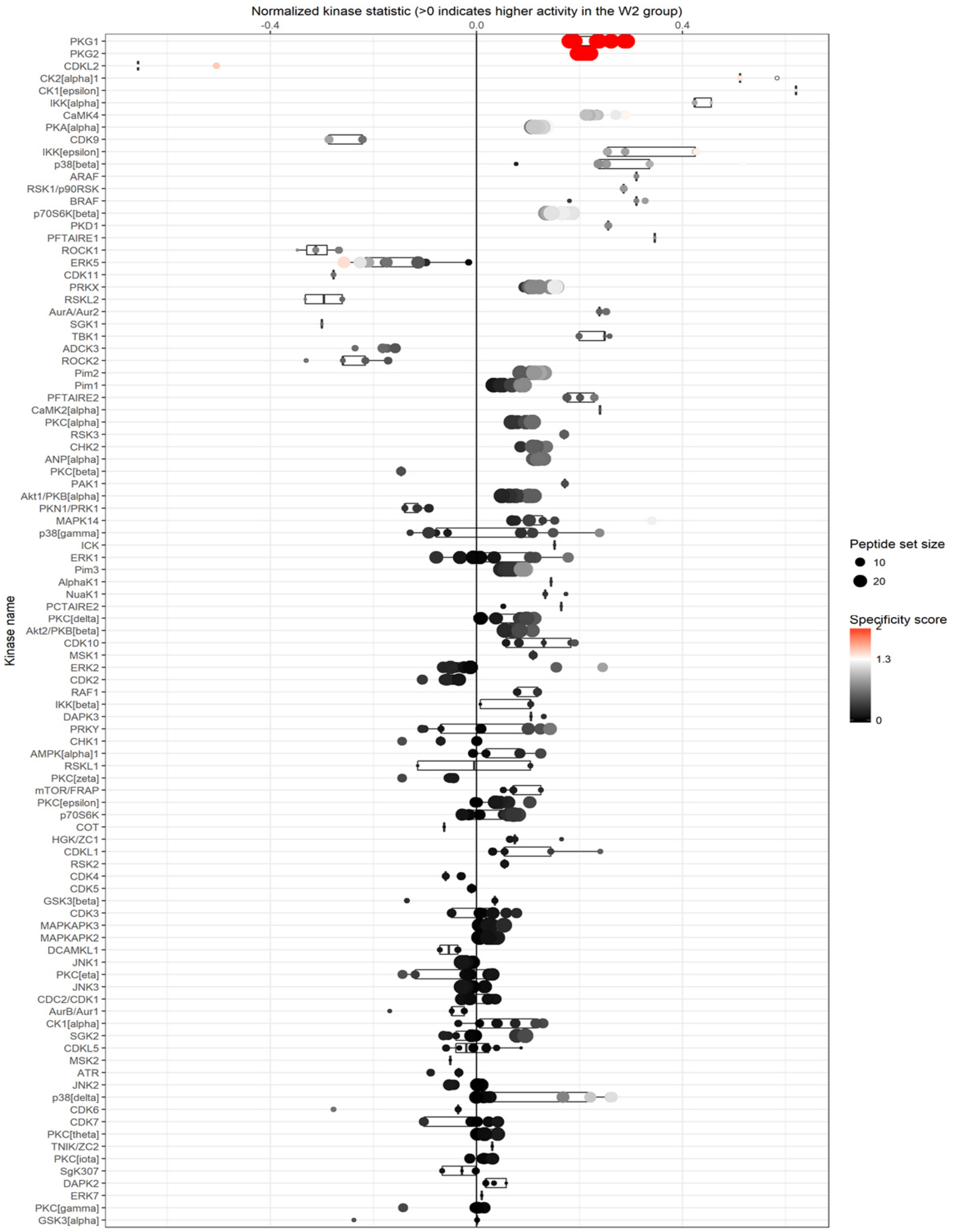

Normalized kinase statistic (>0 indicates higher activity in the W2 group)
-0.4
0.0
0.4
Kinase name
PKG1
PKG2
CDKL2
CK2[alpha]1
CK1[epsilon]
IKK[alpha]
CaMK4
PKA[alpha]
CDK9
IKK[epsilon]
p38[beta]
ARAF
RSK1/p90RSK
BRAF
p70S6K[beta]
PKD1
PFTAIRE1
ROCK1
ERK5
CDK11
PRKX
RSKL2
AurA/Aur2
SGK1
TBK1
ADCK3
ROCK2
Pim2
Pim1
PFTAIRE2
CaMK2[alpha]
PKC[alpha]
RSK3
CHK2
ANP[alpha]
PKC[beta]
PAK1
Akt1/PKB[alpha]
PKN1/PRK1
MAPK14
p38[gamma]
ICK
ERK1
Pim3
AlphaK1
NuaK1
PCTAIRE2
PKC[delta]
Akt2/PKB[beta]
CDK10
MSK1
ERK2
CDK2
RAF1
IKK[beta]
DAPK3
PRKY
CHK1
AMPK[alpha]1
RSKL1
PKC[zeta]
mTOR/FRAP
PKC[epsilon]
p70S6K
COT
HGK/ZC1
CDKL1
RSK2
CDK4
CDK5
GSK3[beta]
CDK3
MAPKAPK3
MAPKAPK2
DCAMKL1
JNK1
PKC[eta]
JNK3
CDC2/CDK1
AurB/Aur1
CK1[alpha]
SGK2
CDKL5
MSK2
ATR
JNK2
p38[delta]
CDK6
CDK7
PKC[theta]
TNIK/ZC2
PKC[iota]
SgK307
DAPK2
ERK7
PKC[gamma]
GSK3[alpha]
Peptide set size
10
20
Specificity score
2
1.3
0
