# Supplementary material for: Ruxolitinib mediated paradoxical JAK2 hyperphosphorylation is due to the protection of activation loop tyrosines from phosphatases
Source: Leukemia. 2025 Apr 23;39(7):1678–91. doi: 10.1038/s41375-025-02594-7 (PMC12208895; doi:10.1038/s41375-025-02594-7)
Supplement: Supplementary file 6 — MPN patients Ruxo vs non-JAK inhibitor treatment [file 41375_2025_2594_MOESM6_ESM.pdf]

MLL ID	Therapy	Entity	*PIM1*	*PIM2*	*TGM2*	*ID1*	*MPL*	*GEM*	*SERPINA1*
MLL_10200	Ruxolitinib (J	PMF	6,76	6,73	4,36	1,79	0,95	-1,14	7,50
MLL_10214	Ruxolitinib (J	PMF	6,34	6,21	2,42	0,54	2,56	-2,64	7,75
MLL_10216	Ruxolitinib (J	PMF	6,83	8,20	2,70	1,83	0,72	-3,19	8,93
MLL_10218	Ruxolitinib (J	PMF	7,75	6,46	5,09	1,10	-0,36	0,86	3,92
MLL_10223	Ruxolitinib (J	PMF	5,24	6,79	0,80	1,65	3,51	-2,33	4,38
MLL_10227	Ruxolitinib (J	PMF	7,07	6,24	4,06	1,55	-0,09	-2,57	7,15
MLL_15379	Ruxolitinib (J	PV	7,88	6,69	3,89	2,73	0,62	-1,09	5,36
MLL_17849	Ruxolitinib (J	PMF	6,82	6,25	4,52	1,63	2,12	1,85	6,88
MLL_17976	Ruxolitinib (J	PMF	7,35	5,75	6,65	3,48	1,15	-1,65	6,07
MLL_18218	Ruxolitinib (J	PV	7,25	6,67	4,71	2,02	0,15	-1,88	6,63
MLL_18230	Ruxolitinib (J	PMF	6,13	6,93	2,42	2,35	1,02	-1,12	7,60
MLL_18233	Ruxolitinib (J	PMF	7,17	8,12	2,82	0,75	0,62	-1,45	8,60
MLL_18548	Ruxolitinib (J	PV	6,94	6,63	3,62	2,50	1,33	2,31	6,79
MLL_18556	Ruxolitinib (J	PMF	6,56	7,96	3,03	1,34	1,75	-0,18	8,30
MLL_18561	Ruxolitinib (J	PV	6,49	4,90	4,10	0,26	-0,75	-2,46	6,59
MLL_18562	Ruxolitinib (J	PV	5,62	5,47	3,23	0,99	-0,99	-0,99	6,45
MLL_18563	Ruxolitinib (J	PV	7,01	7,43	3,47	1,33	1,06	0,34	8,35
MLL_18565	Ruxolitinib (J	ET	5,75	4,88	4,97	0,82	0,71	-1,29	5,41
MLL_18566	Ruxolitinib (J	PMF	6,92	6,83	3,32	2,88	1,53	-0,91	8,34
MLL_19200	Ruxolitinib (J	PMF	6,77	6,75	3,87	0,64	1,65	0,22	7,30
MLL_19688	Ruxolitinib (J	PMF	7,36	7,96	4,50	2,15	0,82	-0,62	7,88
MLL_19729	Ruxolitinib (J	PMF	7,51	6,72	4,69	2,51	1,79	1,74	6,91
MLL_20181	Ruxolitinib (J	PMF	6,93	7,21	1,94	1,20	0,28	0,36	7,95
MLL_20188	Ruxolitinib (J	PMF	7,19	6,55	4,10	1,14	1,42	1,42	6,59
MLL_20193	Ruxolitinib (J	PV	6,03	5,42	4,38	1,57	1,66	0,72	6,37
MLL_20374	Ruxolitinib (J	PMF	5,78	7,34	1,98	0,06	1,26	0,40	8,28
MLL_20404	Ruxolitinib (J	PV	8,23	6,84	4,94	2,92	1,68	-0,56	6,57
MLL_20599	Ruxolitinib (J	PMF	7,71	6,55	4,13	1,98	1,66	2,53	6,40
MLL_21381	Ruxolitinib (J	PV	7,87	6,44	4,88	1,83	-0,82	-0,48	8,81
MLL_30022	Ruxolitinib (J	PMF	7,69	6,20	4,96	1,95	2,94	-2,08	6,92
MLL_54455	Ruxolitinib (J	ET	8,05	6,79	4,74	2,41	2,47	-0,42	7,30
MLL_55542	Ruxolitinib (J	PMF	8,66	9,60	2,71	2,74	0,73	-1,46	8,87
MLL_55549	Ruxolitinib (J	PV	7,53	6,16	4,21	0,74	-0,69	-0,80	6,94
MLL_55550	Ruxolitinib (J	ET	7,27	5,75	4,07	0,78	-0,15	0,39	5,88
MLL_55673	Ruxolitinib (J	PV	6,84	5,07	4,44	-1,38	-1,07	-0,36	6,24
MLL_55867	Ruxolitinib (J	ET	6,08	6,40	2,46	1,99	0,99	-0,24	7,53
MLL_10160	Other	PMF	8,67	7,98	7,10	1,42	-0,64	-1,84	6,94
MLL_10161	Other	PMF	7,32	7,16	4,29	1,46	0,14	-1,53	8,39
MLL_10162	Other	PMF	7,40	7,11	5,17	2,05	0,31	1,76	6,73
MLL_10169	Other	PMF	8,54	7,54	6,75	1,70	0,35	-2,31	7,18
MLL_10170	Other	PMF	8,54	6,89	5,07	1,19	0,96	-2,54	5,71
MLL_10172	Other	PMF	6,99	7,72	4,70	1,38	-0,06	-0,70	8,10
MLL_10178	Other	PMF	9,11	6,25	6,34	0,43	0,77	-1,06	5,17
MLL_10181	Other	PMF	7,22	6,74	5,00	2,74	3,34	-0,68	6,19
MLL_10188	Other	PMF	7,09	6,63	5,76	-0,15	2,00	0,83	7,20
MLL_10189	Other	PMF	6,81	8,14	2,33	2,02	1,87	-1,10	7,51
MLL_10191	Other	PMF	7,41	6,99	3,85	1,65	-0,05	-1,40	7,63
MLL_10198	Other	PMF	5,38	6,96	1,40	0,91	1,28	-2,46	7,78
MLL_10199	Other	PMF	6,26	6,65	4,12	1,62	0,63	-1,44	7,02

MLL_10204	Other	PMF	6,99	6,01	3,99	1,25	0,15	-0,33	6,99
MLL_10211	Other	PMF	9,10	7,11	5,78	1,17	0,74	-0,29	7,82
MLL_12490	Other	PV	7,16	5,83	4,80	2,01	0,13	0,54	6,55
MLL_13900	Other	PV	6,64	6,71	3,38	1,26	-0,38	-0,25	7,17
MLL_13973	Other	PV	7,69	7,07	4,63	1,61	-1,47	2,72	8,96
MLL_13979	Other	ET	5,82	4,98	2,98	1,01	0,28	-0,40	5,46
MLL_13981	Other	PV	7,66	5,29	5,06	1,27	-0,37	1,51	6,59
MLL_13982	Other	PV	6,86	5,63	3,42	1,55	0,48	0,75	6,20
MLL_14115	Other	PV	7,55	6,75	4,49	1,52	-0,92	-1,93	9,37
MLL_14221	Other	PV	7,54	5,96	4,10	1,29	-0,97	-0,50	8,29
MLL_14445	Other	PV	7,88	5,91	5,29	1,72	0,75	1,21	6,67
MLL_14753	Other	PV	7,65	6,25	4,88	2,03	-1,08	0,31	7,21
MLL_16779	Other	PV	7,05	5,61	4,18	2,35	-0,41	0,71	6,09
MLL_17845	Other	PMF	7,03	5,73	7,30	0,35	1,13	3,97	8,49
MLL_17847	Other	PV	6,08	5,58	4,21	1,16	-0,12	-1,80	5,99
MLL_17853	Other	PMF	5,66	6,45	1,69	0,14	1,78	0,55	6,40
MLL_17857	Other	PV	6,92	6,06	4,44	1,75	-0,40	-1,73	6,63
MLL_17858	Other	PV	5,83	6,32	3,30	0,42	-1,20	2,69	6,64
MLL_17859	Other	PV	5,96	6,22	5,53	0,75	-0,07	-1,71	7,13
MLL_17967	Other	PV	5,46	6,06	2,42	3,69	0,87	-1,24	7,78
MLL_17971	Other	PV	6,36	6,18	3,05	1,91	-0,26	1,79	6,70
MLL_17979	Other	ET	5,90	5,13	3,03	1,58	0,48	-0,69	5,77
MLL_18225	Other	PV	6,63	5,71	3,99	1,04	-0,29	-1,01	6,86
MLL_18227	Other	PV	5,99	6,21	3,32	1,48	-0,45	1,08	5,29
MLL_18551	Other	ET	6,06	5,69	3,47	1,21	-0,28	-1,23	6,14
MLL_18557	Other	ET	6,46	5,46	4,19	1,13	-0,86	-0,46	4,20
MLL_18558	Other	ET	6,80	5,85	3,72	1,90	0,22	1,53	6,12
MLL_18560	Other	PMF	7,39	8,42	2,91	0,87	0,38	0,10	7,55
MLL_18564	Other	ET	5,68	6,01	3,34	1,72	0,55	1,54	4,76
MLL_18567	Other	PV	8,43	6,51	5,43	1,84	-1,12	0,08	7,62
MLL_18797	Other	PMF	6,37	5,97	3,41	0,73	1,64	-2,32	6,76
MLL_18798	Other	PMF	6,89	7,75	4,12	1,66	1,96	-0,39	8,26
MLL_19183	Other	PMF	8,37	8,47	5,44	1,95	-0,61	0,22	7,77
MLL_19187	Other	PMF	6,55	6,46	2,29	2,38	1,21	1,31	7,39
MLL_19191	Other	PMF	6,52	6,68	3,54	1,79	-0,30	1,33	7,02
MLL_19291	Other	ET	6,26	6,09	1,28	0,40	0,40	3,03	5,77
MLL_19400	Other	ET	6,08	5,99	2,94	1,73	2,93	1,67	7,24
MLL_19478	Other	PMF	7,35	7,42	3,22	1,99	-0,32	1,25	5,91
MLL_19547	Other	PV	6,15	6,48	4,91	1,94	-0,57	1,87	5,29
MLL_19556	Other	ET	6,00	4,99	2,49	2,01	0,10	1,55	5,67
MLL_19679	Other	PV	7,32	6,29	4,28	0,73	-0,70	1,02	7,82
MLL_19683	Other	PV	6,67	5,72	4,19	2,01	-0,40	-1,17	7,71
MLL_19692	Other	PV	6,51	6,00	3,71	0,87	-0,15	-0,74	6,06
MLL_19698	Other	PV	5,94	6,15	2,32	1,15	-0,10	1,91	7,12
MLL_19723	Other	PMF	7,71	6,37	5,35	0,80	-0,22	0,55	8,02
MLL_19731	Other	PMF	7,91	6,31	4,95	2,54	-0,29	-0,10	6,44
MLL_19865	Other	PV	6,65	5,82	4,15	1,71	-0,51	-0,78	6,76
MLL_19866	Other	ET	6,02	6,59	2,28	0,15	0,46	-0,23	6,97
MLL_19870	Other	PV	7,44	5,84	4,84	1,83	1,13	0,89	6,05
MLL_19877	Other	ET	5,80	6,06	2,46	2,18	0,60	0,79	7,37

MLL_19978	Other	PV	6,33	5,17	2,93	2,22	-0,54	1,31	5,65
MLL_19981	Other	PV	7,73	6,65	4,51	0,95	0,94	0,26	7,07
MLL_20183	Other	PMF	5,72	5,21	2,60	0,09	0,09	-0,36	7,39
MLL_20196	Other	PV	5,52	4,86	4,49	1,80	0,23	-0,17	5,49
MLL_20386	Other	PV	7,13	4,88	4,24	1,18	-0,39	-2,08	6,21
MLL_20387	Other	ET	5,21	5,76	2,60	1,53	0,10	-2,35	6,23
MLL_20391	Other	PV	5,98	5,79	3,46	2,21	-0,61	-0,39	6,70
MLL_20409	Other	PMF	6,87	6,29	4,08	1,06	0,56	-2,07	8,39
MLL_20410	Other	PV	4,98	4,22	2,65	0,41	0,78	-1,50	5,72
MLL_20412	Other	PV	8,21	5,91	4,78	1,73	-0,08	-0,45	6,25
MLL_20603	Other	PV	7,78	7,57	4,39	2,32	0,53	1,50	5,97
MLL_21269	Other	PMF	6,45	6,62	1,88	2,82	1,95	-0,28	8,38
MLL_21519	Other	PV	6,69	5,57	4,27	0,62	1,02	-0,56	5,65
MLL_22173	Other	PV	7,13	5,88	4,82	1,41	-0,38	-2,00	6,94
MLL_54435	Other	PV	8,06	7,25	7,56	2,36	-0,70	-1,11	6,87
MLL_54614	Other	PMF	8,08	6,44	5,32	1,65	1,71	-1,56	7,61
MLL_54616	Other	PMF	8,90	8,72	5,68	1,26	-1,67	1,01	8,71
MLL_54620	Other	PMF	6,83	6,52	3,98	2,56	1,59	-0,99	6,90
MLL_54621	Other	PMF	7,90	8,28	3,98	3,04	0,96	-1,35	8,72
MLL_54623	Other	PMF	7,98	8,51	3,81	1,70	1,43	-1,57	8,37
MLL_54624	Other	ET	6,46	5,10	4,29	-0,17	0,26	-1,66	5,40
MLL_55310	Other	PMF	7,65	7,09	5,07	1,51	0,49	-2,98	8,67
MLL_55518	Other	ET	6,20	5,51	2,07	1,17	-0,43	2,67	6,85
MLL_55522	Other	ET	7,43	7,64	2,87	2,17	2,84	0,90	8,06
MLL_55535	Other	ET	7,56	5,05	3,78	0,73	-0,02	0,05	4,88
MLL_55537	Other	PV	7,83	5,99	4,59	1,20	0,22	-0,87	6,36
MLL_55538	Other	ET	6,19	5,27	2,36	-0,07	-1,41	1,92	5,02
MLL_55540	Other	ET	7,10	5,67	4,24	1,59	0,52	0,09	4,91
MLL_55541	Other	ET	6,72	6,36	3,84	1,59	0,46	1,00	4,54
MLL_55543	Other	ET	5,60	5,41	3,74	1,65	0,22	-2,46	6,19
MLL_55551	Other	ET	6,15	5,95	2,97	1,63	0,20	-1,27	6,42
MLL_55552	Other	ET	7,39	6,50	4,60	2,54	2,81	-0,38	7,95
MLL_55642	Other	PMF	7,26	6,99	3,87	0,67	0,61	-2,30	6,37
MLL_55670	Other	PV	7,56	5,88	4,13	0,64	0,49	1,85	7,43
MLL_55675	Other	PV	8,55	6,70	4,16	0,49	-1,60	-0,25	7,36
MLL_55677	Other	PV	8,05	7,19	5,41	0,07	-0,42	1,48	8,05
MLL_55681	Other	PV	7,75	7,01	4,18	1,05	0,82	1,47	7,66
MLL_55682	Other	PV	7,55	5,48	3,93	0,63	0,44	0,44	5,67
MLL_55683	Other	PV	7,72	6,40	4,59	2,39	1,35	-0,04	7,29
MLL_55684	Other	PV	7,97	6,05	4,45	1,80	-0,12	-1,11	7,81
MLL_55685	Other	PV	7,75	6,39	4,51	-0,05	-0,57	-1,24	7,72
MLL_55686	Other	ET	6,78	5,42	3,46	2,08	0,34	0,82	5,68
MLL_55687	Other	ET	7,26	6,90	3,62	1,37	0,30	0,95	7,67
MLL_55688	Other	PV	7,05	6,14	4,32	1,98	-0,94	0,05	6,61
MLL_55689	Other	PV	7,27	5,53	3,83	2,04	0,36	-0,65	6,37
MLL_55690	Other	ET	7,93	7,23	5,21	2,15	1,61	0,57	7,05
MLL_55691	Other	PV	7,28	6,23	3,57	1,67	-0,56	-1,57	7,97
MLL_55852	Other	PV	7,59	6,91	4,03	1,83	0,30	2,21	7,37
MLL_55853	Other	PV	6,72	4,86	3,74	0,83	0,67	-0,66	5,66
MLL_55855	Other	PV	7,24	6,67	3,90	0,78	1,11	-0,19	7,93

MLL_55856	Other	PV	7,52	6,32	4,19	0,58	-1,23	-2,84	7,86
MLL_55857	Other	PV	7,53	6,98	3,71	2,46	0,95	0,16	8,59
MLL_55858	Other	PV	7,83	6,39	4,68	2,44	1,23	-0,42	7,86
MLL_55859	Other	PV	8,19	7,14	3,72	1,49	-1,17	0,48	7,51
MLL_55861	Other	PV	9,01	5,68	7,24	3,55	-0,55	-2,43	3,34
MLL_55862	Other	ET	6,18	5,67	2,41	0,38	0,41	0,14	7,10
MLL_55863	Other	PV	7,25	5,14	4,70	1,65	-1,60	-0,64	5,74
MLL_55864	Other	PV	6,57	5,52	4,55	0,91	-0,99	-2,14	6,92
MLL_55865	Other	ET	6,20	5,32	3,26	1,89	0,65	-0,86	6,07
MLL_55866	Other	PV	6,78	5,95	4,24	2,04	0,68	-1,40	6,49
